# Tumor-suppressive circRHOBTB3 is excreted out of cells via exosome to sustain colorectal cancer cell fitness

**DOI:** 10.1186/s12943-022-01511-1

**Published:** 2022-02-11

**Authors:** Chaoyi Chen, Hongfei Yu, Fengyan Han, Xuan Lai, Kehong Ye, Siqin Lei, Minglang Mai, Maode Lai, Honghe Zhang

**Affiliations:** 1 Department of Pathology and Women’s Hospital, Zhejiang University School of Medicine, Research Unit of Intelligence Classification of Tumor Pathology and Precision Therapy, Chinese Academy of Medical Sciences (2019RU042), Hangzhou, 310058 China; 2 Department of Pathology, Research Unit of Intelligence Classification of Tumor Pathology and Precision Therapy of Chinese Academy of Medical Sciences (2019RU042), Zhejiang University School of Medicine, Hangzhou, 310058 China; 3 Key Laboratory of Disease Proteomics, Hangzhou, 310058 Zhejiang Province China; 4 Cancer Center, Zhejiang University, Hangzhou, 310058 China; 5 Department of Pharmacology, China Pharmaceutical University, Nanjing, 210009 China

**Keywords:** Exosome, Metastasis, Colorectal cancer, circRHOBTB3, Circularization

## Abstract

**Background & Aims:**

To clarify the biological roles, circularization process and secretion pathway of circRHOBTB3 in colorectal cancer (CRC) progression.

**Methods:**

We performed a comprehensive analysis of circRNA levels in serum exosomes from multiple types of cancer patients in public databases and verified the higher level of circRHOBTB3 in CRC sera versus healthy donors by RT-qPCR. Then, the function of circRHOBTB3 in CRC was investigated in vitro and in vivo. RNA-seq and RNA pull-down assays together with mass spectrometry identified the downstream signals and the binding proteins of circRHOBTB3. Finally, Antisense oligonucleotides (ASOs) were designed to target circularization and secretion elements of circRHOBTB3 for CRC therapy.

**Results:**

circRHOBTB3 levels were increased in the sera but was downregulated in tissue samples in CRC, and the downregulation was associated with poor prognosis. Furthermore, circRHOBTB3 acts a tumor-suppressive circRNA by repressing metabolic pathways, intracellular ROS production in CRC. Several key elements were discovered to regulate circRHOBTB3 circularization and exosomal secretion. Moreover, SNF8 was identified that sorts circRHOBTB3 into exosomes. Interestingly, we found that CRC cells could actively secrete more circRHOBTB3 than normal cells. According to the sequence of regulatory elements for circularization and exosomal secretion, we designed and synthesized ASOs, which increased circRHOBTB3 expression and blocked circRHOBTB3 exosomal secretion. More importantly, ASOs could inhibit CRC growth and metastasis in vitro and in vivo.

**Conclusions:**

circRHOBTB3 plays a tumor-suppressive role in CRC and has to be excreted out of cells to sustain cancer cell fitness. ASOs targeting regulatory elements for circularization and exosomal secretion will become a novel antitumor strategy.

**Supplementary Information:**

The online version contains supplementary material available at 10.1186/s12943-022-01511-1.

## Introduction

Circular RNAs (circRNAs) comprise a large class of noncoding RNAs without a 5′ terminal cap and a 3′ terminal poly (A) tail that are produced by RNA polymerase II transcription through back-back splicing to form covalently closed RNA circles [1]. Along with the development of next-generation sequencing technology, especially the application of rRNA-free sequencing analysis, a large number of circRNAs have been discovered in eukaryotes. The process of circRNA splicing is regulated by multiple factors, including the transcriptional elongation rate, multiple cis-trans regulatory sites, splicing complexes, and cis- or trans-acting elements [2]. Recent advances implicate these novel circRNAs in various biological processes, such as cell proliferation, adhesion, apoptosis and survival [3]. Otherwise, some disease-related circRNAs have also been identified in cardiovascular diseases, neurological disorders, metabolic syndromes and tumor progression [3], which function by sponging specific microRNAs as competitive endogenous RNAs (ceRNAs), binding proteins, regulating RNA transcription and splicing, and even producing peptides through translation.

To date, circRNAs have gained substantial attention in the cancer research field. Although an increasing number of studies have reported aberrant circRNAs in breast, lung and colorectal cancer [4], the biological function, molecular mechanism and circularization patterns of circRNAs in tumor cells are still unclear. Interestingly, circRNAs can be secreted from cancer cells into the circulation through exosomes with unknown pathological functions [5]. Exosomes are 30–150 nm extracellular vesicles produced by a variety of cells, and their cargos include proteins, lipids and nucleic acids. To date, endosomal sorting complex required for transport (ESCRT)-dependent and ESCRT-independent pathways have been reported to regulate exosomes to sort cargos. However, the ESCRT-II complex might be involved in the exosomal sorting process for nucleic acids, and the specific sequences of miRNAs could determine whether the cargoes are sorted into exosomes [6]. Compared with linear RNAs, circRNAs are more easily sorted into exosomes [7], but the exosome-specific sorting mechanism for circRNAs remains completely unknown.

circRHOBTB3 was produced by splicing and circularization of the host gene RHOBTB3. As a Rho GTPase-family ATPase, the host gene RHOBTB3 is involved in membrane transport [8, 9] and proteasomal degradation [10] and can inhibit tumor growth by promoting the proteasomal degradation of HIF-α in renal cancer [11]. Previous studies also showed circRHOBTB3 as a tumor-suppressive circRNA in ovarian cancer [12], stomach cancer [13], CRC [14] and hepatocellular carcinoma (HCC) [15]. However, the circularization patterns and exosomal secretion pathway are not defined. In this study, we not only clarified the suppressive roles of circRHOBTB3 in CRC but also suggested a novel “exosomal escape” hypothesis, which suggests that tumor cells have to excrete suppressive circRNAs to maintain cancer cell fitness.

Antisense oligonucleotides (ASOs) are 8–50 nt single-stranded oligo (deoxy) nucleotides or (deoxy) nucleotide analogs that can be artificially synthesized with a variety of modifications, such as phosphorothioate. ASOs can regulate RNA biosynthesis, protein translation, alternative splicing and the interaction of RNA-binding proteins (RBPs) with RNA [16]. Along with the advancement of technology, these modified ASOs have been applied to treat some diseases, such as cytomegalovirus (CMV)-induced chorioretinitis, Duchenne muscular dystrophy (DMD), homozygous familial hypercholesterolemia (HoFH), acute hepatic porphyria (AHP), and spinal muscular atrophy (SMA) [16, 17]. Here, we investigated the potential therapeutic effect of specific ASOs targeting circRHOBTB3 negative circularization and exosomal sorting elements in CRC, which could become a novel and promising targeting strategy for antitumor therapy.

## Materials and methods

### Public datasets analysis

The total-RNA expression profiling of serum exosomes from 12 CRC patients, 21 HCC patients, 32 PAAD patients, and 14 healthy donors of GSE100063, GSE100206, GSE100207, GSE100232 were obtained from GEO database. All these datasets were conducted on GPL11154 platform (Illumina HiSeq 2000). Processed data were downloaded from GEO and re-analyzed using MeV 4.9.0 software, *p* < 0.05 and Foldchange> 1.2 were considered statistically significant. The TCGA data were downloaded from The Human Protein Atlas (https://www.proteinatlas.org/about/download).

### Clinical materials

Colorectal carcinoma and paired normal tissues samples (*n* = 35), another CRC cohort (*n* = 69) were obtained from patients during operation at the Department of Medicine, Sir Run Run Shaw Hospital, Zhejiang University School of Medicine. 16 healthy donor serum samples and 18 CRC serum samples were obtained from CRC patients as well as medical examiners at the Department of Medicine (Ethics Committee number: 2021–021), Sir Run Run Shaw Hospital, Zhejiang University School of Medicine. All participating patients were informed. Patients or the public were not involved in the design, or conduct, or reporting, or dissemination plans of our research.

### Quantifications and statistical analysis

Data in this paper are presented as mean ± SD or mean ± SEM, and paired sample or two independent sample Student’s t test was used to test for significant differences between two groups. Kaplan–Meier survival analysis was performed using the software IBM SPSS Statistics 20 with the Log-rank test. *P*-values < 0.05 were considered statistically significant, *P < 0.05, **P < 0.01, ***P < 0.001, NS, not significant.

All other materials and methods can be found in supplementary materials.

## Results

### circRHOBTB3 is highly expressed in exosomes but downregulated in tumor tissues

**Fig. 1:**
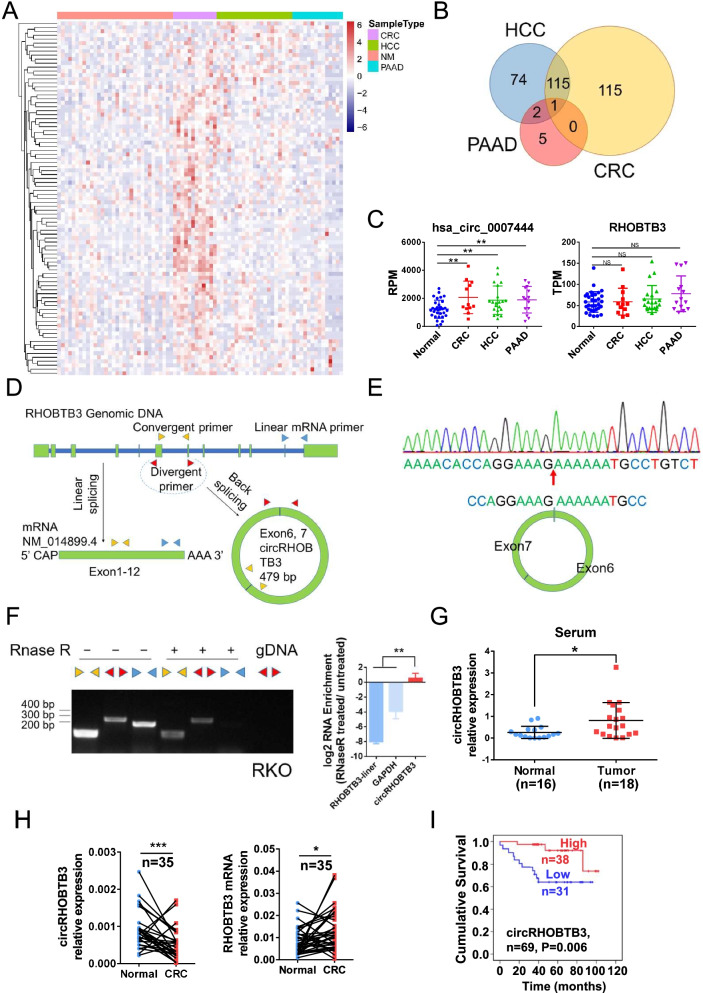
Identification and characterization of circRHOBTB3 in CRC. (**A**) Heatmap of differentially expressed circRNAs in sera from CRC, HCC, and PAAD patients compared with healthy donors. (**B**) Venn diagram of significantly upregulated circRNAs in CRC, HCC, and PAAD serum samples. (**C**) Expression of hsa_circ_000744 and its host gene in serum samples from CRC, HCC, PAAD patients and healthy donors. (**D**) Sketch map and primers for circRHOBTB3. (**E**) Sanger sequencing for confirmation of the specific back-splicing site of circRHOBTB3. (**F**) RNase R treatment, RT-PCR and RT-qPCR for confirmation of circRHOBTB3. (**G**) Detection of circRHOBTB3 in serum samples from CRC patients by RT-qPCR (levels normalized to those of GAPDH). (**H**) Detection of circRHOBTB3 and RHOBTB3 in paired normal and CRC tissue in the verification cohort by RT-qPCR (levels normalized to those of GAPDH). (**I**) Kaplan-Meier survival analysis of CRC patients according to the expression level of circRHOBTB3 using the log-rank test (*n* = 69). All experiments were repeated for three times, data were shown as mean ± SD, * *P* < 0.05, ** *P* < 0.01, *** *P* < 0.001, NS *P* > 0.05, in Student’s test (**C, F, G**), or paired Student’s test (**H**)

First, we reanalyzed the data of 4 GEO datasets (GSE100063, GSE100206, GSE100207, GSE100232), including total RNA-seq data of serum exosomes from 12 CRC patients, 21 HCC patients, 32 pancreatic adenocarcinoma (PAAD) patients, and 14 healthy donors, in the same sequencing platform (Fig. S1A). The results showed that a number of circRNAs were differentially expressed in tumor patient serum exosomes (Fig. 1A). However, only the circular RNA hsa_circ_0007444 was upregulated in all three types of cancer-derived exosomes (Fig. 1B). Although there was a high level of hsa_circ_0007444, the level of its host gene RHOBTB3 was not increased in these three types of cancer-derived exosomes (Fig. 1C). In The Cancer Genome Atlas (TCGA) database, the host gene RHOBTB3 was also not significantly increased in these three types of cancer tissues compared to normal tissues, and its expression did not correlate significantly with prognosis (Fig. S1B).

Because hsa_circ_0007444 consists of exons 6 and 7 of RHOBTB3, we hereafter referred to it as circRHOBTB3. To determine the existence and circular characteristics of circRHOBTB3, we designed specific divergent primers for detecting circRHOBTB3, specific linear mRNA primers for detecting RHOBTB3 and convergent primers for detecting both circRHOBTB3 and RHOBTB3 (Fig. 1D). Then, the specific back-splicing site of circRHOBTB3 was identified by RT-PCR and Sanger sequencing (Fig. 1E). Compared with the linear host genes RHOBTB3 and GAPDH, circRHOBTB3 significantly resisted RNase R treatment (Fig. 1F). Then, we detected the expression of circRHOBTB3 in serum from 18 CRC patients and 16 healthy donors. The results showed a higher circRHOBTB3 level in the serum of CRC patients than in that of healthy donors, which was consistent with the database (Fig. 1G). Unexpectedly, circRHOBTB3 was significantly downregulated in CRC tissues, while the host gene RHOBTB3 was significantly upregulated (Fig. 1H). Furthermore, we detected the expression of circRHOBTB3 in a cohort of 69 CRC tissue samples, which showed that patients with a higher level of circRHOBTB3 displayed better overall survival (Fig. 1I). Taken together, these clinical data demonstrated a high level of circRHOBTB3 in the serum exosomes of CRC patients, which contrasted with the low expression of circRHOBTB3 in CRC tumor tissues.

### circRHOBTB3 is a tumor-suppressive circRNA and secreted outside of tumor cells

**Fig. 2:**
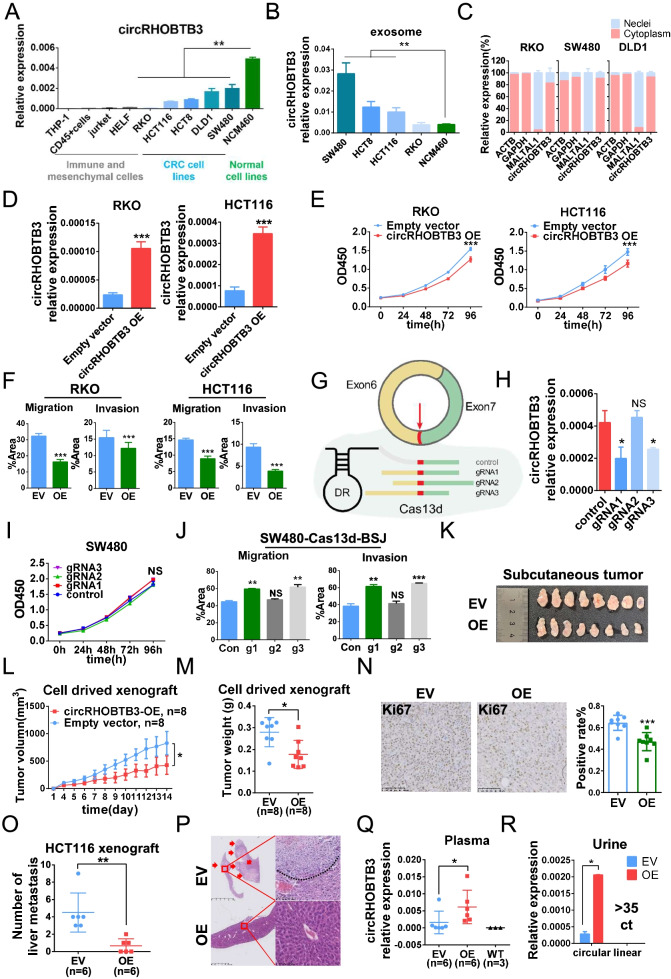
circRHOBTB3 is secreted outside of tumor cells and suppresses CRC cells in vitro and in vivo. (**A**) Expression of circRHOBTB3 in CRC or normal colonic epithelial cell lines and (**B**) exosomes derived from them. (**C**) Nucleoplasmic RNA isolation and RT-qPCR for the localization of circRHOBTB3. ACTB and GAPDH were used as cytoplasmic positive controls, and MALTAL1 was used as a nuclear positive control. (**D**) Overexpression of circRHOBTB3 by lentivirus vector in RKO and HCT116 cells. The quantification analysis results of (**E**) cell proliferation, (**F**) migration and invasion assays of circRHOBTB3-OE RKO and HCT116 cells. (**G**) Schematic of gRNA design and construction for circRHOBTB3. (**H**) Specific KD of circRHOBTB3 by RfxCas13d-BSJ-gRNA in SW480 cells. The quantification analysis results of (**I**) Cell proliferation, (**J**) migration and invasion assays of circRHOBTB3 KD SW480 cells. (**K**) Images of the subcutaneous xenograft tumors. (**L**) Tumor volume and (**M**) tumor weight of xenograft tumors. (**N**) Ki67 staining of xenograft tumors. The right column diagram shows the quantification analysis results. (**O**) The quantification of the liver distant metastatic foci of liver distant metastasis model established by splenic injection of control (empty vector) and circRHOBTB3-OE HCT116 cells in nude mice. (**P**) HE staining in liver metastatic foci. (**Q**) Expression of circRHOBTB3 in the plasma and (**R**) urine of mice inoculated with control or circRHOBTB3-OE HCT116 cells. Data were shown as mean ± SD (A, B, C, D, E, H, I, L, M, N, O, Q, X) or mean ± SEM (F, J), * *P* < 0.05, ** *P* < 0.01, *** *P* < 0.001, NS *P* > 0.05, in Student’s test (A, B, C, D, E, H, I, L, M, N, O, Q, R) or paired Student’s test (F, J)

To determine the source of circRHOBTB3 in serum exosomes, we detected circRHOBTB3 expression in CRC cell lines (RKO, HCT116, HCT8, DLD1 and SW480), a normal intestinal epithelial cell line (NCM460), a fibroblast cell line (HELF), immune-related cell lines (THP-1 and Jurkat), and CD45^+^ cells from a healthy donor. The results showed relatively high circRHOBTB3 expression in NCM460 cells, moderate expression in CRC cell lines, and low expression in fibroblasts, immune-related cell lines and CD45^+^ cells (Fig. 2A). From these data, we could infer that circRHOBTB3 in serum exosomes of CRC patients might be derived from tumor cells but not from stromal cells. Then, exosomes were extracted from the culture supernatant of RKO, SW480, HCT8 and HCT116 cells (Fig. S2A), which were identified by detecting TSG101 and CD63 (Fig. S2B). Interestingly, the circRHOBTB3 level was lowest in the exosomes derived from NCM460 cells, although it had the highest circRHOBTB3 expression (Fig. 2B). In addition, we also examined the localization of circRHOBTB3 in CRC cell lines (RKO, SW480 and DLD1) and found that circRHOBTB3 was mainly localized in the cytoplasm (Fig. 2C). Then, we overexpressed circRHOBTB3 in RKO, HCT116, HCT8 and DLD1 cells (Fig. 2D and S3A), which showed that circRHOBTB3 could inhibit cell proliferation (Fig. 2E and S3B), migration and invasion (Fig. 2F and S3C). Moreover, the overexpression of circRHOBTB3 did not change the expression of the host gene RHOBTB3 (Fig. S3D). Even RHOBTB3 knockdown in RKO cells did not change the migration and invasion potential (Fig. S3E). These results demonstrated that circRHOBTB3 plays tumor suppressive roles in CRC independent of the regulation of its host gene RHOBTB3. To further verify the function of circRHOBTB3 in CRC, we tried to design siRNAs at the back-splicing junction site of circRHOBTB3, but unfortunately, all siRNAs displayed off-target effects (Fig. S4A), so we used CRISPR/RfxCas13d-BSJ-gRNA technology [18] to specifically knock down circRHOBTB3 (Fig. 2G). As shown in Fig. 2H, gRNA1 and gRNA3 effectively knocked down circRHOBTB3, but gRNA2 did not. None of the gRNAs affected the expression of the host gene RHOBTB3 in SW480 cells (Fig. S4B). CircRHOBTB3 knockdown did not change the proliferation potential (Fig. 2I) but promoted SW480 cell migration and invasion (Fig. 2J and S4C).

The data above identified circRHOBTB3 as a tumor-suppressive circRNA in vitro. However, the roles of circRHOBTB3 in vivo remain unknown. Therefore, we constructed xenograft tumor models by subcutaneously injecting circRHOBTB3-overexpressing HCT116 cells into nude mice. CircRHOBTB3 overexpression (circRHOBTB3-OE) significantly inhibited xenograft tumor growth (Fig. 2K and S4D). The tumor volume (Fig. 2L) and weight (Fig. 2M) were significantly reduced in the circRHOBTB3-OE group compared with the empty vector (EV) group. Ki67 staining assays showed that circRHOBTB3 expression inhibited CRC cell proliferation in vivo (Fig. 2N). Then, we inoculated these CRC cells into the spleens of nude mice to observe the phenotype of liver metastasis, which showed that there were fewer liver metastatic foci in the circRHOBTB3-OE group than in the EV group according to gross observation (Fig. 2O and S4E) and HE staining (Fig. 2P). Interestingly, human circRHOBTB3 could be detected in the plasma from xenograft tumor mouse models but not in the plasma from wild-type mice; moreover, the circRHOBTB3-OE group presented higher levels of circRHOBTB3 in plasma than the EV group (Fig. 2Q). More intriguingly, circRHOBTB3 could also be detected in the mouse urine of both groups and was significantly higher in the circRHOBTB3-OE group than in the EV group. As a negative control, however, the host gene RHOBTB3 was not detectable in the urine of either group of mice (Fig. 2R). These results indicated that circRHOBTB3 inhibited CRC growth and metastasis in vivo; circRHOBTB3 was packed into exosomes and secreted into circulation and eventually excreted through the urinary system. According to these in vitro and in vivo data, we presumed that CRC cells have to excrete tumor-suppressive circRHOBTB3 to maintain the characteristics of cancer invasion and metastasis through exosome secretion.

### The circularization of circRHOBTB3 is controlled by regulator elements

**Fig. 3:**
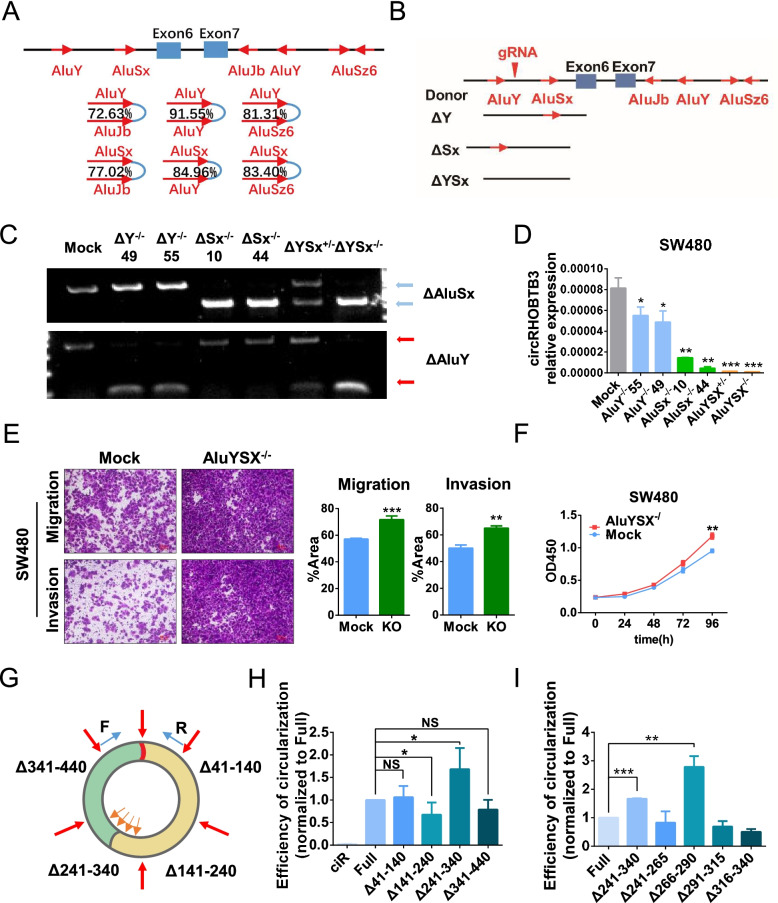
The biogenesis of circRHOBTB3 is regulated by a cis-element on its own sequence. (**A**) Schematic diagram of the genomic regions of RHOBTB3 exon 6 and exon 7 containing flanking Alu repeats and long introns. (**B**) Schematic diagram of the gRNA and homologous recombination donors. (**C**) RT-PCR and (**D**) RT–qPCR for CRISPR/Cas9-mediated genomic deletions in SW480 cells. (**E**) Cell migration, invasion, (**F**) and proliferation assays of circRHOBTB3-KO SW480 cells. The right column diagram shows the quantification analysis results. (**G**) Schematic diagram of truncated circRHOBTB3 vector construction and primer design for detection. (**H**) Circularization efficiency of 100 nt truncation and (**I**) further 25 nt truncation, normalized to FULL. All experiments were repeated for three times, data were shown as mean ± SD (**D, F**) or mean ± SEM (E, H, I), * *P* < 0.05, ** *P* < 0.01, *** *P* < 0.001, NS *P* > 0.05, in Student’s test (**D, F**) or paired Student’s test (**E, H, I**)

The biogenesis of circRNAs is complex and poorly understood, and has three possible mechanisms: spliceosome-dependent biogenesis, cis-element-facilitated circRNA formation and RNA binding protein (RBP)-regulated circRNA formation [19]. Short interspersed elements (SINEs) have been identified as the key regulatory sequences flanking intron that mediate circRNA circularization [20, 21]. Therefore, we analyzed the intron sequences of circRHOBTB3, which showed that there were six SINE elements in the introns flanking circRHOBTB3, and these sites composed six pairs of complementary pairing patterns (Fig. 3A). To identify the key cis-element of circRHOBTB3 circularization, we deleted AluY or AluSx through the CRISPR/Cas9 system (Fig. 3B) to produce AluY-deficient, AluSx-deficient and AluY-AluSx-deficient cells (Fig. 3C). When either the AluY or AluSx element was deleted, the expression of circRHOBTB3 was significantly reduced; however, deletion of AluY and AluSx at the same time completely abolished the circularization ability of circRHOBTB3 (Fig. 3D). Otherwise, AluSx exhibited a stronger effect on regulating circRHOBTB3 circularization than AluY, which might be attributed to steric hindrance. These results suggest that there are cis-elements in the introns that regulate the biogenesis of circRHOBTB3. Consistently, deletion of both AluY and AluSx led to circRHOBTB3 knockout (KO) in SW480 cells, which not only promoted the migration and invasion (Fig. 3E) but also increased cell proliferation potential (Fig. 3F).

**Fig. 4:**
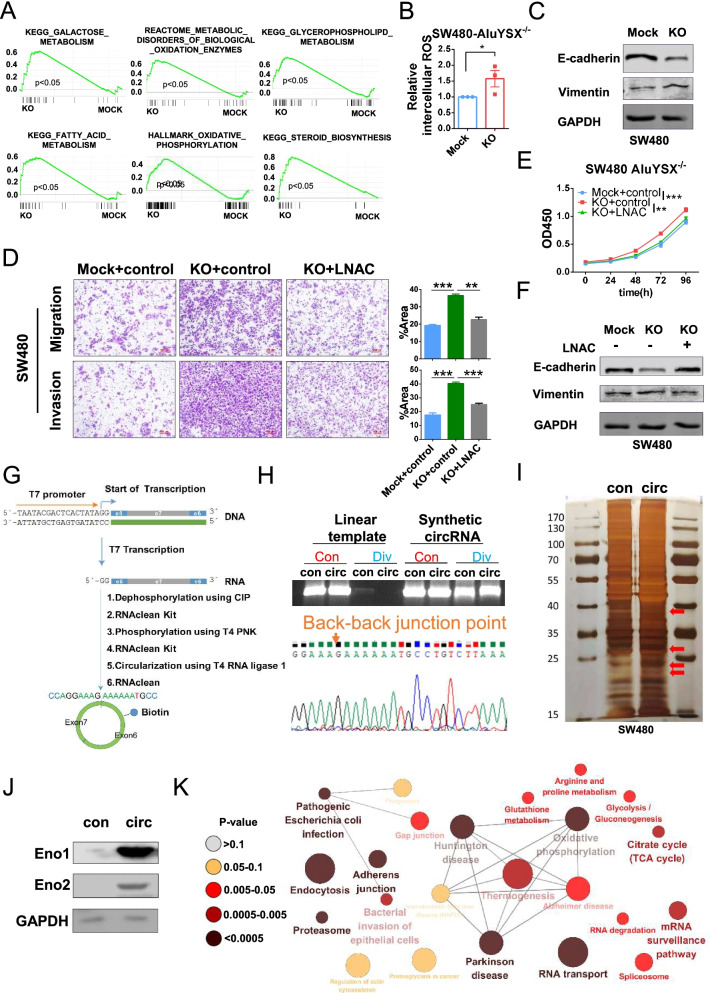
circRHOBTB3 inhibits EMT by binding to metabolism-related proteins to regulate ROS. (**A**) GSEA and (**B**) ROS levels of circRHOBTB3-KO SW480 cells compared with mock SW480 cells. (**C**) Western blot of mock and circRHOBTB3-KO SW480 cells using anti-E-cadherin and anti-vimentin antibodies. (**D**) Cell migration, invasion, and (**E**) proliferation assays and (**F**) Western blot of control and LNAC-treated circRHOBTB3-KO SW480 cells. The right column diagram shows the quantification analysis results. (**G**) Schematic diagram of biotin-labeled circular RNA synthesis. (**H**) Verification of the synthesized biotin-labeled circRHOBTB3. (**I**) Different protein bands detected by silver staining for mass spectrometry. (**J**) Western blot of RNA pull-down products using anti-Eno1 and anti-Eno2 antibodies. Anti-GAPDH were used as negative control. (**K**) ClueGO functional analysis of potential proteins interacting with circRHOBTB3. All experiments were repeated for three times, data were shown as mean ± SD (**E**) or mean ± SEM (**B**, **D**), * *P* < 0.05, ** *P* < 0.01, *** P < 0.001, NS *P* > 0.05, in Student’s test (**E**) or paired Student’s test (**B, D**)

The biogenesis of circRNA is also considered a special alternative splicing event [22–24] that can be regulated by cis-elements in exons. Therefore, we wanted to determine whether there are also cis-elements in the exon sequence of circRHOBTB3 itself. For this, we cloned a series of truncated circRHOBTB3 expression vectors and transfected them into 293 T cells to examine the circularization efficiency of circRHOBTB3 (Fig. 3G). As shown in Fig. 4H, deletion of the 141–240 nt region in the exon of circRHOBTB3 significantly reduced the circularization efficiency of circRHOBTB3, while deletion of the 241–340 nt region significantly increased circRHOBTB3 circularization efficiency (Fig. 3H), which demonstrated that there were cis-elements in the 141–240 nt and 241–340 nt regions that promote or inhibit circRHOBTB3 circularization, respectively. Furthermore, the 266–290 nt motif was identified as the key cis-element in the 214–340 nt region that negatively regulates circRHOBTB3 circularization (Fig. 3I). These data suggest that the biogenesis of circRHOBTB3 is regulated by cis-elements not only in the introns flanking region but also in its exon sequence.

### circRHOBTB3 inhibits epithelial-mesenchymal transition (EMT) by regulating ROS and metabolism

To investigate how circRHOBTB3 functions the suppressive effect on CRC, we analyzed and predicted the protein-coding potential of circRHOBTB3, which showed that there were an IRES element and an overlapping open reading frame (Fig. S5A and S5B). Recently, some studies have reported that circRNAs with overlapping ORF might encode some new proteins through multiple rounds of translation [25]. Hence, we inserted a segment of the FLAG coding sequence into the circRHOBTB3 expression vector (Fig. S5C), but no obvious protein was detected in circRHOBTB3-overexpressing cells (Fig. S5D), indicating that circRHOBTB3 might not have protein coding ability.

Next, we performed RNA-seq in circRHOBTB3-KO SW480 cells and analyzed the normalized data by gene set enrichment analysis (GSEA) [26], which showed that circRHOBTB3 KO caused alterations in multiple metabolic pathways, including galactose, fatty acid, and glycerophospholipid metabolism; oxidative phosphorylation; steroid biosynthesis and biological oxidation (Fig. 4A). These results indicated that circRHOBTB3 could regulate metabolic pathways. In addition, the adherent junction and G2/M checkpoint pathways, which are associated with migration, invasion and cell cycle arrest, were downregulated. The NF-KB, RAS and ERK signaling pathways were also significantly upregulated by circRHOBTB3 KO (Fig. S5E). Differentially expressed genes (DEGs) functional and signaling pathway enrichment analysis with the Database for Annotation, Visualization, and Integrated Discovery (DAVID) showed that the upregulated genes were also enriched in metabolism, proliferation and EMT pathways (Fig. S5F). To validate the RNA-seq data, we performed RT-qPCR to detect the representative genes. Consistently, the expression of the DEGs related to metabolism was significantly upregulated in circRHOBTB3-KO cells, whereas the expression of these genes was significantly downregulated in circRHOBTB3 overexpression RKO cells. As expected, re-expression of circRHOBTB3 in circRHOBTB3-KO SW480 cells decreased the expression of these metabolism-related genes (Fig. S6A). Previous studies have reported that metabolic disorder could cause the intracellular accumulation of ROS and promote EMT [27, 28]. We used a DCFH-DA probe to detect ROS levels, and the results showed that ROS levels were significantly increased in circRHOBTB3-KO cells versus SW480 Mock cells (Fig. 4B). Immunoblotting assays showed that the epithelial marker E-cadherin was downregulated, but the mesenchymal marker vimentin was upregulated in circRHOBTB3-KO cells (Fig. 4C).

Moreover, overexpression of circRHOBTB3 in circRHOBTB3-KO cells not only inhibited proliferation (Fig. S6B), migration and invasion (Fig. S6C) but also rescued the EMT phenotype (Fig. S6D). Interestingly, the intracellular ROS level was also significantly reversed (Fig. S6E). Then, we used the ROS scavenger N-acetyl-L-cysteine (LNAC) to treat circRHOBTB3-KO cells (Fig. S6F), and the results showed that the depletion of intracellular ROS partially inhibited migration and invasion (Fig. 4D) and reversed the proliferation effect (Fig. 4E) and EMT phenotype (Fig. 4F). To further clarify the mechanism by which circRHOBTB3 plays a tumor suppressive role by regulating metabolic pathways, we synthesized biotin-labeled circRHOBTB3 in vitro (Fig. 4G) and verified the sequence of circRHOBTB3 by RT-PCR and Sanger sequencing (Fig. 4H). Then, RNA pull-down assays were performed to identify the proteins binding to circRHOBTB3 in SW480 cells (Fig. 4I). Furthermore, LS/MS was applied to identify the specific circRHOBTB3 binding proteins (Fig. S7A and S7B), which were confirmed by immunoblotting (Fig. 4J). Interestingly, these proteins were also enriched in metabolism-, RNA processing and transport-, and adhesion-related pathways (Fig. 4K and S7C). Taken together, these data suggest that circRHOBTB3 might regulate intracellular ROS levels to inhibit tumor cell proliferation and EMT by interacting with metabolic enzymes such as ENO1 and ENO2.

### circRHOBTB3 is sorted into exosomes and secreted outside of tumor cells by the specific motif interacting with SNF8

**Fig. 5:**
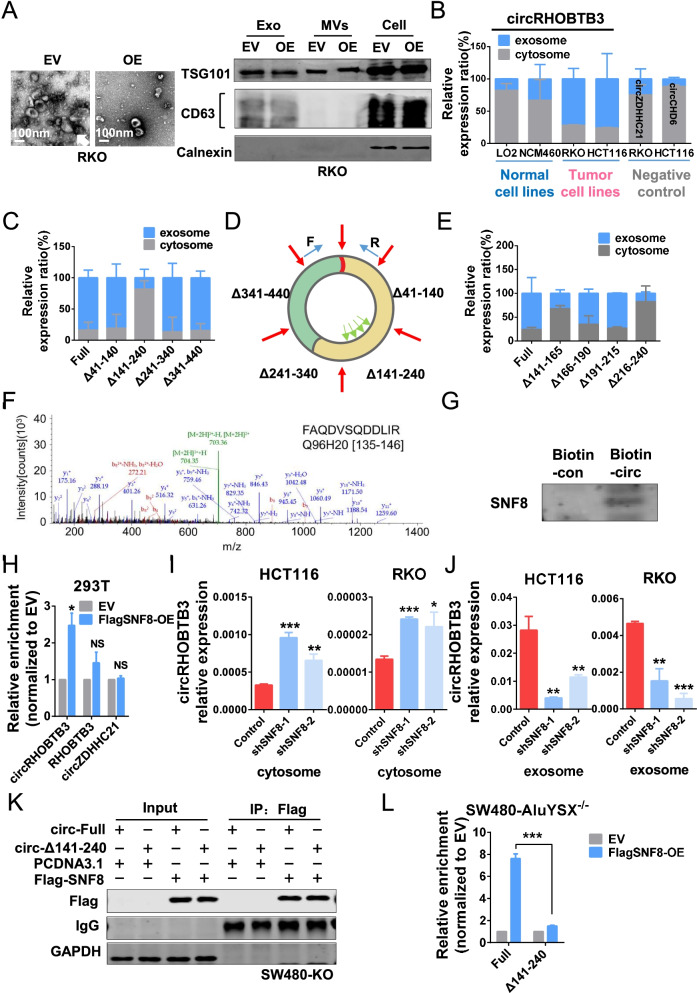
circRHOBTB3 is sorted into exosomes according to its specific motif sequence. (**A**) Observation of CRC cell exosomes by transmission electron microscopy (TEM) and Western blotting with anti-CD63 and anti-TSG101 antibodies. Anti-calnexin was used as a negative control, and cellular lysates were used as positive loading controls. (**B**) Exosomal secretion assay of normal cell lines, tumor cell lines and (**C**) RKO cells transfected with truncated circRHOBTB3 vector. (**D**) Schematic diagram of truncated circRHOBTB3 vector construction and primer design for exosomal secretion assay. (**E**) Exosomal secretion assay of RKO cells transfected with 25 nt truncated circRHOBTB3 vector. (**F**) The specific amino acid sequence of SNF8 shown by second-order mass spectrometry. (**G**) WB of RNA pull-down products confirmed the interaction of circRHOBTB3 with SNF8. (**H**) RT-qPCR of the anti-FLAG RIP assay. RHOBTB3 and circZDHHC21 were used as negative controls. (**I**) Expression of circRHOBTB3 in the cytosome and (**J**) exosomes of SNF8-KD HCT116 and RKO cells. (**K**) Western blot and (**L**) RT-qPCR of anti-SNF8 RIP assay in full-length and Δ141–240 circRHOBTB3 re-expressed SW480-KO cells. All experiments were repeated for three times, data were shown as mean ± SD (**I, J**) or mean ± SEM (**B, C, E, H, L**), * *P* < 0.05, ** *P* < 0.01, *** *P* < 0.001, NS P > 0.05, in Student’s test (**I, J**) or paired Student’s test (**H, L**)

To test the hypothesis that the tumor-suppressive circRNA was actively pumped out from cancer cells by exosome secretion, we transfected the circRHOBTB3 expression vector into 293 T cells, which showed higher circRHOBTB3 levels in the cell lysate but not in the culture medium (Fig. S8A). This result indicated that 293 T cells, as a relatively normal cell line, could not secrete circRHOBTB3 through exosomes. Intriguingly, circRHOBTB3 overexpression in RKO cells led not only to an increase in intracellular circRHOBTB3 but also to a significantly higher circRHOBTB3 level in culture medium, which demonstrated that the CRC cell line RKO could secrete more circRHOBTB3 than 293 T cells. To further verify our observation, we transfected the circRHOBTB3 expression vector into the normal intestinal epithelial cell line NCM460, normal liver cell line LO2, and CRC cell lines RKO and HCT116; then, we isolated exosomes to detect the efficiency of circRHOBTB3 secretion (Fig. 5A). As shown in Fig. 5B, circRHOBTB3 was retained in the cytoplasm of normal cell lines NCM460 and LO2 but excreted out of tumor cells such as RKO and HCT116 along with exosomes. Another two circular RNAs, circZDHHC21 and circCHD6, functioning as pro-oncogenes (data not shown), were not enriched in exosomes from RKO and HCT116 cell culture medium. These data suggest that tumor cells might specifically eliminate unfavorable circRNAs via exosome secretion.

The process by which nucleic acids are incorporated into exosomes remains unclear. Recent studies reported that miRNAs were sorted into exosomes according to their specific motif sequence [29], which suggests that some specific regulatory elements could facilitate RNA entry into exosomes. To investigate the key element in circRHOBTB3 that regulates exosome sorting, we detected circRHOBTB3 exosome secretion efficiency in RKO cells transfected with the four truncated circRHOBTB3 expression vectors described above. CircRHOBTB3 was no longer enriched in exosomes when the 141–240 nt sequence was missing (Fig. 5C). By further constructing a truncated circRHOBTB3 expression vector (Fig. 5D), we found that deletion of the 141–165 or 216–240 nt sequence significantly blocked exosomal secretion of circRHOBTB3 (Fig. 5E). These results demonstrate that the cis-elements in circRHOBTB3 itself could regulate the process of circRHOBTB3 secretion by exosomes.

The endosomal system regulates the biogenesis of exosomes via ESCRT-dependent and ESCRT-independent mechanisms, while the ESCRT-II subcomplex, as an RNA binding complex, may sort RNA to exosomes [6]. Among those circRHOBTB3-binding proteins, we fortunately found that SNF8 was a member of the ESCRT-II complex interacting with circRHOBTB3 (Fig. 5F). Moreover, the interaction between circRHOBTB3 and SNF8 was validated by RNA pull-down assay and immunoblotting (Fig. 5G). To further confirm their interaction, we performed an RNA immunoprecipitation (RIP) assay using an anti-flag antibody in SNF8-flag-overexpressing cells (Fig. S8B), which showed that SNF8 could bind to circRHOBTB3 but not to the mRNA of the host gene RHOBTB3 or to circZDHHC21 (Fig. 5H). We also performed an endogenous RIP assay using an SNF8 antibody in SW480 cells and obtained the same results (Fig. S8C). Interestingly, when SNF8 was knocked down using siRNAs in HCT116 cells (Fig. S8D), the intracellular circRHOBTB3 level was increased (Fig. S8E). Then, we produced stable SNF8 knockdown HCT116 and RKO cells by shRNA transfection (Fig. S8F). Consistently, SNF8 knockdown increased the expression of circRHOBTB3 in cell lysates (Fig. 5I) but decreased the expression of circRHOBTB3 in exosomes (Fig. 5J). The host genes RHOBTB3 and circZDHHC21 were not changed (Fig. S8G and S8H). These data demonstrated that SNF8 could carry circRHOBTB3 into exomes, which were sequentially secreted outside of tumor cells. Our previous data identified that the 141–240 nt region of circRHOBTB3 is the key element that regulates circRHOBTB3 exosome secretion. Therefore, we performed an exogenous RIP assay in circRHOBTB3-KO SW480 cells to investigate whether SNF8 could bind to the 141–240 nt region of circRHOBTB3 (Fig. 5K). The results showed that SNF8 could pull-down exogenous full-length circRHOBTB3 but not circRHOBTB3 without the 141–240 nt region (Fig. 5L). Together with previous data, we proposed a novel circRNA exosome secretion mechanism: circRHOBTB3 is specifically sorted into exosomes via the interaction between the ESCRT-II complex member SNF8 and its own specific element and is secreted outside of tumor cells. Here, we call this the “tumor exosomal escape mechanism”.

### Specific ASOs inhibit tumor progression by controlling circRHOBTB3 circularization and secretion

**Fig. 6:**
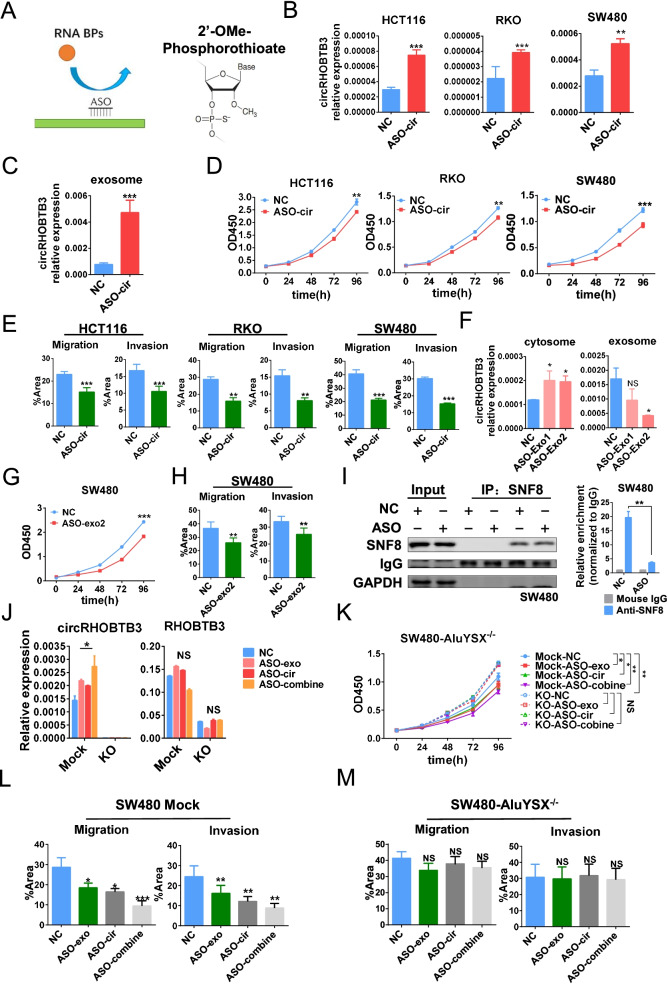
ASO inhibits CRC cell proliferation, migration and invasion *in vitro* by controlling circRHOBTB3 circularization and secretion. (**A**) Schematic of the functional mechanism and chemical modification of ASO. (**B**) Expression of circRHOBTB3 in ASO-NC- and ASO-cir-treated HCT116, RKO and SW480 cells. (**C**) Expression of circRHOBTB3 in ASO-NC- and ASO-cir-treated HCT116 cells. (**D**) Cell proliferation and (**E**) quantification of migration and invasion assays of ASO-NC- and ASO-cir-treated HCT116, RKO and SW480 cells. (**F**) Expression of circRHOBTB3 in the cytosome and exosomes of ASO-NC-, ASO-exo1-, and ASO-exo2-treated SW480 cells. (**G**) Cell proliferation and (**H**) quantification of migration and invasion assays of ASO-NC- and ASO-exo2-treated SW480 cells. (**I**) Western blotting and RT-qPCR of the anti-SNF8 RIP assay in ASO-NC- and ASO-exo-treated SW480 cells. (**J**) Relative circRHOBTB3 or RHOBTB3 expression and (**K**) cell proliferation and (**L, M**) quantification of cell migration and invasion assay of ASO-NC, ASO-exo, ASO-cir and ASO-cir combined with ASO-exo treated SW480 (**L**) Mock and (**M**) KO cells. All experiments were repeated for three times, data were shown as mean ± SD (**B, C, D, F, G,J, K**) or mean ± SEM (**E, H, I, L, M**), * *P* < 0.05, ** *P* < 0.01, *** *P* < 0.001, NS *P* > 0.05, in Student’s test (**B, C, D, F, G,J, K**) or paired Student’s test (**E, H, I, L, M**)

Our previous data identified circRHOBTB3 as a tumor-suppressive circRNA that was secreted outside of cells by exosomes. Therefore, we wanted to determine whether it could become a therapeutic strategy by increasing circRHOBTB3 circularization and decreasing secretion. ASOs were designed to bind to RNA untranslated regions to sequester RNA-binding protein recognition by steric hindrance [16]. Then, we designed specific second-generation ASOs with a phosphorothioate backbone and 2ʹ-O-substituted oligoribonucleotide segments (Fig. 6A) targeting a negative circularization element (ASO-cir for 266–290 nt) and exosome secretion elements (ASO-exo1 for 141–165 nt and ASO-exo2 for 216–240 nt) to increase the intracellular level of circRHOBTB3 (Fig. S9A). When ASO-cir was transfected into HCT116, RKO and SW480 cells, the expression level of intracellular circRHOBTB3 was significantly elevated (Fig. 6B), but the host gene RHOBTB3 was not obviously changed in HCT116 and RKO cells but decreased in SW480 cells (Fig. S9B). ASO-cir also increased the level of circRHOBTB3 in cell culture exosomes, which indicated that increasing circRHOBTB3 was also sorted into exosomes and secreted from tumor cells along with enhanced circRHOBTB3 circularization by ASO-cir (Fig. 6C). As expected, ASO-cir not only inhibited cell proliferation (Fig. 6D) but also decreased the migration and invasion potential (Fig. 6E and S9C) of HCT116, RKO and SW480 cells. Moreover, ASO-cir also promoted RKO cell apoptosis (Fig. S9D).

Then, we tested the effect of ASO-exo1 and ASO-exo2, which target exosome secretion elements on CRC cells. As shown in Fig. 6F, both ASO-exo1 and ASO-exo2 increased the intracellular level of circRHOBTB3; ASO-exo2 blocked circRHOBTB3 exosome secretion, but ASO-exo1 did not, in SW480 cells. Therefore, we used ASO-exo2 to investigate the antitumor effect, which showed that ASO-exo2 could significantly repress not only the proliferation (Fig. 6G) but also the migration and invasion of SW480 cells (Fig. 6H and S9E). Furthermore, RIP assays were performed after transfection of ASO-exo2 into SW480 cells, which showed that the binding of SNF8 to circRHOBTB3 was disrupted by ASO-exo2 and demonstrated that ASO-exo2 (hereafter called ASO-exo) inhibited the exosomal sorting of circRHOBTB3 by blocking the interaction between SNF8 and circRHOBTB3 (Fig. 6I).

Although ASO-cir promoted circRHOBTB3 circularization and expression, upregulated circRHOBTB3 excretion from tumor cells was still observed. Therefore, we evaluated the specificity and antitumor effect of ASO-cir combined with ASO-exo on CRC. As shown in Fig. 6J, the combination of ASO-cir with ASO-exo treatment led to a higher intracellular level of circRHOBTB3 than ASO-cir or ASO-exo separate treatment. Interestingly, the combination of ASO-cir with ASO-exo treatment significantly enhanced the suppressive effect not only on proliferation (Fig. 6K) but also on migration and invasion (Fig. 6L and S9F) in SW480 mock cells. However, these phenotypes caused by ASO-cir, ASO-exo or their combination were not observed in circRHOBTB3-KO cells (Fig. 6M and S9F). These data demonstrated that both ASO-cir and ASO-exo might play an antitumor role by specifically targeting circRHOBTB3.

**Fig. 7:**
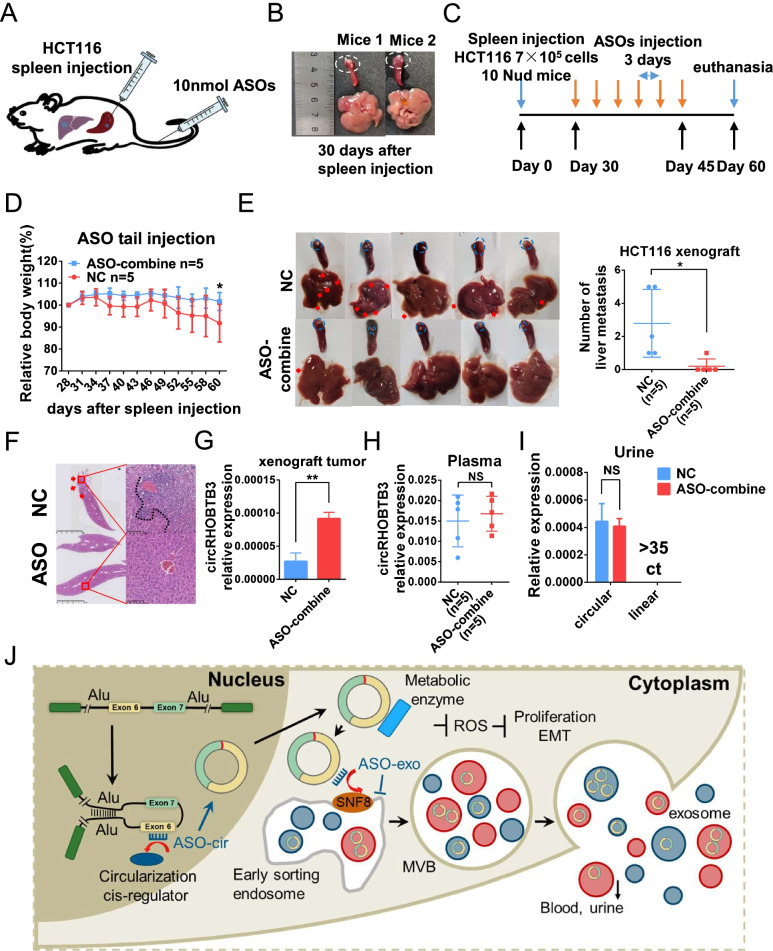
ASO inhibits CRC cell growth and metastasis in vivo. (**A**) Schematic of the liver metastasis model for ASO injection construction. Twenty nmol ASO-NC or 10 nmol ASO-exo combined with 10 nmol ASO-cir was used for tail vein injection. (**B**) Images of the spleen primary (the blue circle indicates primary foci) and liver. (**C**) Schematic of ASO tail vein injection. (**D**) Body weight of mice after ASO tail vein injection. (**E**) Images of the liver distant metastasis model established by splenic injection of HCT116 cells (the red arrows indicate metastatic foci). The right scatter plot shows the quantification analysis results. (**F**) HE staining in liver metastatic foci. (**G**) Expression of circRHOBTB3 in xenograft tumors, (**G**) plasma, and (**I**) urine with tail vein injection of ASO-combined or ASO-NC. (**J**) Schematic model of the mechanism by which ASO blocks circRHOBTB3 circulation and exosomal sorting. Data were shown as mean ± SD (**D, E, G , H , I**), * *P* < 0.05, ** *P* < 0.01, NS *P* > 0.05, in Student’s test (**D, E, G , H , I**)

To further investigate the antitumor effect of the ASOs in vivo and to explore the potential of clinical application, we constructed a mouse hepatic metastasis model through spleen injection with CRC cells (Fig. 7A). Thirty days after spleen inoculation of HCT116 cells, we euthanized the mice and confirmed that primary tumors developed in the spleen without any obvious liver metastatic foci, which confirmed that ASOs were injected before liver metastasis occurred (Fig. 7B). Then, we injected the control and a mixture of ASO-exo and ASO-cir through the tail vein every three days. Thirteen days after the sixth treatment, the mice were euthanized, and the liver, spleen, plasma, and urine were collected (Fig. 7C). Interestingly, the weight of mice in the control group was significantly decreased, but there was no change in the treatment group, which suggested that combination treatment with ASO-exo and ASO-cir could significantly reduce the cachexia of tumors (Fig. 7D). As expected, gross observation (Fig. 7E) and HE staining (Fig. 7F) showed fewer liver metastatic foci in the ASO treatment group than in the control group. Moreover, these ASOs could significantly elevate circRHOBTB3 expression in spleen primary foci (Fig. 7G), while the level of circRHOBTB3 was not changed in serum and urine, suggesting that ASOs could reduce the excretion of circRHOBTB3 into plasma (Fig. 7H) and urine (Fig. 7I). Taken together, these data indicated that ASO-cir and ASO-exo could be applied as a novel remedy for CRC therapy that regulates circularization and exosomal sorting.

## Discussion

Tumor initiation and progression remain poorly understood despite the evidence that this kind of somatic evolutionary process was driven by the accumulation of genetic alterations [30]. However, recent studies showed that there were similar mutation patterns and overall mutational burdens between primary tumors and metastatic foci, which demonstrated no metastasis driver mutations in the tumor progression process [31]. Therefore, epigenetic and transcriptional regulation are critical drivers of tumor metastasis. Recently, circRNAs have become important epigenetic regulatory molecules in cancer development [32, 33]. Here, we showed that circRHOBTB3 functions as a tumor suppressive molecule in CRC by regulating metabolic pathways and intracellular reactive oxygen species (ROS) levels. During our ongoing project, Chen et al. reported that circRHOBTB3 exerted suppressive effects on CRC aggressiveness through the HuR/PTBP1 axis [14]. Moreover, circRHOBTB3 inhibited ovarian cancer progression by regulating PI3k/AKT signaling pathway [12]. As miRNA sponge, circRHOBTB3 repressed gastric cancer progression by miR-654-3p/p21 axis [13]. And it could also block miR-18a maturation to function as a suppressive role in liver cancer [15]. In addition, circRHOBTB3 could regulate autophagy via miR-600/NACC1 axis in pancreatic ductal carcinoma [34]. However, we found a large discrepancy between circRHOBTB3 expression in tumor tissues and in serum exosomes. Furthermore, we proposed a novel tumor escape theory in which tumor cells have to excrete tumor-suppressive circRHOBTB3 through exosomes to sustain cancer cell fitness.

Although we clarified that circRHOBTB3 could inhibit CRC progression by repressing EMT and interacting with the metabolic enzymes ENO1 and ENO2, it is still a great challenge for us to target tumor-suppressive circRNAs for CRC therapy. It is conceivable that increasing intracellular circRHOBTB3 levels might achieve the expected antitumor effect in CRC. To date, circRNA biogenesis has been considered a special splicing event [22–24], and alternative splicing is regulated by a variety of factors, including exon splicing enhancers (ESEs) or exon splicing silencers (ESSs) [35, 36]. The sequential assembly of spliceosomes with small nuclear ribonucleoproteins (snRNPs) catalyzes the ligation reaction of the 5′ donor site downstream of the exon with the 3′ acceptor site upstream to produce circRNAs. Otherwise, the cis-elements in flanking introns regulate circRNA circularization through juxtaposing reverse complementary sequences to form RNA duplexes [1, 19, 37]. In these processes, many RBPs, such as FUS, QKI and MBL [38–40], also participate in regulating circRNA formation. In addition to the above regulatory mechanisms, we first reported that the motifs of the circRNAs themselves could also regulate circRNA circularization as cis-elements. Deletion of 241–340 nt substantially increased the circularization efficiency of circRHOBTB3, and the 266–290 nt motif was the key cis-element to negatively regulate circRHOBTB3 circularization. The possible mechanism is that this cis-element acts as an ESS bound to some RBPs to impede spliceosome activity and block circRHOBTB3 circularization. However, we could use this cis-element as an antitumor target by increasing circRHOBTB3 levels. Since the first FDA-approved ASO was applied to treat retinitis caused by opportunistic CMV [17], ASO has been proven to be a new effectual remedy for targeting RNA processing. In particular, second-generation ASOs modified by 2′-O-methyl (2′-OME)-phosphorothioate (PS) were approved by the FDA to treat SMA and DMD [16]. ASOs have become a potential therapeutic strategy for diverse malignant diseases along with chemical modification and delivery vehicle development. In this study, we also designed second-generation ASOs to target the negative circularization elements of circRHOBTB3 to increase circRHOBTB3 expression, which presented a suppressive effect on CRC progression in vitro and in vivo. These data indicate that ASOs could be considered an alternative option for targeting tumor-suppressive circRNAs by promoting their circularization.

As carriers, exosomes mediate intercellular communication in the microenvironment and are involved in EMT, angiogenesis, drug resistance, immunity regulation and other biological processes [6, 41]. Many previous studies found abundant circRNAs in exosomes derived from tumor cells [42–44]. Here, we showed that circRHOBTB3 was enriched in serum exosomes from CRC but downregulated in tumor tissues. Interestingly, compared with normal cells, tumor cells prefer to excrete circRHOBTB3 via exosome secretion, which could facilitate escape from the anticancer effects of circRHOBTB3. The endosomal sorting complex required for the transport (ESCRT) system is composed of ESCRT-0, ESCRT-I, ESCRT-II, ESCRT-III and the Vps4-Vta1 protein complex, which form and transport multivesicular endosomes (MVEs) as a protein machinery in eukaryotic cells. Then, exosomes are secreted during the fusion of MVEs with the plasma membrane. Although how nucleic acids as cargoes are sorted into extracellular vesicles passively or actively remains unclear, miRNAs are sorted into exosomes mainly depending on their specific sequence. Otherwise, the ESCRT­II subcomplex could bind to and mediate miRNA exosomal sorting by tetraspanin-enriched microdomains sequestering argonaute 2 (AGO2) [6]. Our findings demonstrated that SNF8, a key component of the ESCRT-II complex, could bind to circRHOBTB3 and sort it into exosomes by interacting with the specific motif of circRHOBTB3. However, an unresolved question is the destination of circRHOBTB3 secreted by exosomes. Intriguingly, we found that circRHOBTB3 was abundantly enriched in urine. Because the particle diameter of exosomes is 30 nm–100 nm, which is equivalent to the pore diameter of the glomerular filtration membrane whose filtration limit is 100 nm [45], exosomes carrying circRHOBTB3 could be filtered from blood to urine by the glomerulus. Another interesting phenomenon was observed: the host gene RHOBTB3 presented a high transcriptional level to produce adequate RHOBTB3, which might be of great importance for the physiological function of both tumor and normal cells. Along with RHOBTB3 transcription, circRHOBTB3 was inevitably produced in tumor cells, but circRHOBTB3 displayed a strong antitumor effect. Therefore, tumor cells must clean up this kind of circRNA to maintain the characteristics of proliferation, invasion and metastasis, which here was defined as the tumor exosomal escape mechanism (Fig. 7J). The novel theory could also partly explain why some circRNAs in tumors function in an opposite role to host genes. However, the tumor exosomal escape mechanism should still be extensively investigated in the context of other tumor inhibitory molecules, such as miRNAs and proteins.

Previously, we verified that ASOs targeting the circRHOBTB3 negative circularization regulation element could increase circRHOBTB3 expression and repress CRC progression. However, the increased circRHOBTB3 was also partially secreted out of tumor cells, which affected the antitumor effect of ASOs. Therefore, we designed another ASO targeting circRHOBTB3 secretion regulation elements to block the interaction between circRHOBTB3 and SNF8, which could inhibit circRHOBTB3 exosomal secretion and CRC progression. More expectedly, combination with two types of ASOs could significantly enhance their tumor suppressive effect. Our clinical data showed 85.7% (30/35) tumor samples carried with a lower level of circRHOBTB3 than the paired normal samples in CRC patients, and the lower expression of circRHOBTB3 in tumor samples was associated with poorer prognosis. Along with the further confirmation of circRHOBTB3 expression in more CRC patients, these ASOs will become a promising therapeutic strategy in future for CRC as they increase intracellular circRHOBTB3 levels.

## Conclusion

In conclusion, our study not only clarified that circRHOBTB3 represses CRC progression by regulating intercellular ROS and metabolism pathways but also proposes a novel tumor escape theory, the tumor exosomal escape mechanism, in which tumor cells excrete tumor-suppressive circRNAs to sustain cancer cell fitness. There will be important significance for the tumor exosomal escape mechanism to understand and treat cancer. More importantly, ASOs could be applied to regulate tumor-suppressive circRNAs by targeting circularization and secretion to facilitate favorable therapeutic effects for cancer, which could provide a new field for cancer therapy in the future.

## Supplementary Information

**Additional file 1: Fig. S1.** Analysis of expression and survival from the TCGA and GEO databases. (A) The reanalyzed GEO datasets in this study. (B) Kaplan-Meier survival analysis of CRC, PAAD, and HCC patients according to the expression level of RHOBTB3 from the TCGA. All experiments were repeated for three times, data were shown as mean ± SD, NS *P* > 0.05, in Student’s test or log-rank test.

**Additional file 2: Fig. S2.** Verification of exosomes. (A) Observation of exosomes by TEM. (B) Western blotting of CRC cell-derived exosomes using anti-CD63 and anti-TSG101 antibodies. Anti-calnexin was used as a negative control, and cellular lysates were used as positive loading controls.

**Additional file 3: Fig. S3.** circRHOBTB3 inhibits CRC cell proliferation, migration and invasion in vitro. (A) Overexpression of circRHOBTB3 via lentivirus vector transfection in HCT8 and DLD1 cells. (B) Cell proliferation of circRHOBTB3-OE HCT8 and DLD1 cells. (C) migration and invasion assays of circRHOBTB3-OE RKO, HCT116, HCT8 and DLD1 cells. The right column diagram shows the quantification analysis results. (D) Expression of RHOBTB3 in circRHOBTB3-OE RKO, HCT116, HCT8 and DLD1 cells. (E) Cell migration and invasion assay of circRHOBTB3-si RKO cells. The right column diagram shows the quantification analysis results. All experiments were repeated for three times, data were shown as mean ± SD (A, B, D) or mean ± SEM (C, E), * *P* < 0.05, ** *P* < 0.01, *** *P* < 0.001, NS *P* > 0.05, in Student’s test (A, B, D) or paired Student’s test (C, E).

**Additional file 4: Fig. S4.** circRHOBTB3 is secreted outside of tumor cells and suppresses CRC cells in vitro and in vivo. (A) siRNA design for circRHOBTB3. (B) Expression of RHOBTB3 in circRHOBTB3-KD SW480 cells treated with RfxCas13d-BSJ-gRNA. (C) Migration and invasion assays of circRHOBTB3 KD SW480 cells. (D) Images of the subcutaneous xenograft tumors. (E) Images of the liver distant metastasis model established by splenic injection of control (empty vector) and circRHOBTB3-OE HCT116 cells in nude mice (the red arrows indicate metastatic foci). Data were shown as mean ± SD (B), NS P > 0.05, in Student’s test (B).

**Additional file 5: Fig. S5.** The potential protein coding ability of circRHOBTB3. (A) IRES and ORF of circRHOBTB3. (B) Schematic of the potential protein encoded by circRHOBTB3. (C) Schematic of Flag-tagged circRHOBTB3 vector construction. (D) Western blotting of EV and Flag-tagged circRHOBTB3-OE 293 T cells using anti-Flag. (E) GSEA, (F) Kyoto Encyclopedia of Genes and Genomes (KEGG) and gene ontology (GO) enrichment analysis of circRHOBTB3-KO SW480 cells versus mock SW480 cells.

**Additional file 6: Fig. S6.** circRHOBTB3 inhibits EMT through regulating ROS. (A) Heatmap of genes related to metabolism in circRHOBTB3-KO SW480 cells, circRHOBTB3-OE RKO cells, and circRHOBTB3-KO SW480 cells with circRHOBTB3 re-expression. (B) Cell proliferation, (C) migration and invasion assay and (D) Western blot of circRHOBTB3 overexpression in circRHOBTB3-KO SW480 cells. The right column diagram shows the quantification analysis results. (E) Relative intercellular ROS level of circRHOBTB3 re-expression and (F) LNAC-treated circRHOBTB3-KO SW480 cells. All experiments were repeated for three times, data were shown as mean ± SD (B) or mean ± SEM (C, E, F), * *P* < 0.05, ** *P* < 0.01, *** *P* < 0.001, NS *P* > 0.05, in Student’s test (B) or paired Student’s test (C, E, F).

**Additional file 7: Fig. S7.** Analysis of mass spectrometry results. (A) Total ion chromatogram (TIC) of control and syn-circRHOBTB3 pulldown products. (B) Venn diagram of proteins identified from control and syn-circRHOBTB3 pulldown products. (C) Enrichment analysis of potential proteins interacting with circRHOBTB3.

**Additional file 8: Fig. S8.** SNF8 regulates the exosome sorting of circRHOBTB3. (A) Expression of circRHOBTB3 in cell lysis or culture supernatant of 293 T and RKO cells. (B) Western blot of Anti-FLAG RIP assay in 293 T cells. (C) Western blotting and RT-qPCR of the anti-SNF8 RIP assay in SW480 cells. (D) Western blotting of SNF8-KD HCT116 cells transfected with siRNA using anti-SNF8. (E) Expression of circRHOBTB3 in SNF8-KD HCT116 cells by siRNA. (F) RT-qPCR and Western blotting of SNF8-KD HCT116 cells transfected with shRNA. (G) Expression of circRHOBTB3 and (H) negative control circZDHHC21 in the cytosome and exosomes of SNF8-KD HCT116 and RKO cells. All experiments were repeated for three times, data were shown as mean ± SD (E, F, G, H) or mean ± SEM (A, C), * *P* < 0.05, ** *P* < 0.01, *** *P* < 0.001, NS *P* > 0.05, in Student’s test (E, F, G, H) or paired Student’s test (A, C).

**Additional file 9: Fig. S9.** ASO inhibited CRC cell proliferation, migration and invasion in vitro. (A) Schematic of ASO design. (B) Expression of RHOBTB3 in ASO-NC- and ASO-cir-treated HCT116, RKO and SW480 cells. (C) Cell migration and invasion assays of ASO-NC- and ASO-cir-treated HCT116, RKO and SW480 cells. (D) Apoptosis assay of ASO-NC- and ASO-cir-treated RKO cells. The right column diagram shows the quantification analysis results. (E) Cell migration and invasion assays of ASO-NC- and ASO-exo2-treated SW480 cells. (F) Cell migration and invasion assays of ASO-NC-, ASO-exo-, ASO-cir- and ASO-cir combined with ASO-exo-treated SW480 mock and KO cells. All experiments were repeated for three times, data were shown as mean ± SD (B) or mean ± SEM (D), * *P* < 0.05, ** P < 0.01, *** *P* < 0.001, NS *P* > 0.05, in Student’s test (B) or paired Student’s test (D).

**Additional file 10: Table S1.** Oligonucleotide sequences used in this study. **Table S2.** Vectors used in this study. **Table S3.** Antibodies used in this study.

## Data Availability

All data generated or analyzed during this study are included either in this article or in the supplementary information files.

## Abbreviations

CRC: Colorectal cancer
HCC: Hepatocellular carcinoma
PAAD: Pancreatic adenocarcinoma
circRNAs: Circular RNAs
ESCRT: Endosomal sorting complex required for transport
ORF: open reading frame
ASO: Antisense oligonucleotides
EMT: Epithelial-mesenchymal transition
RBPs: RNA-binding proteins
ESEs: Exon splicing enhancers
ESSs: Exon splicing silencers; snRNPs: Small nuclear ribonucleoproteins
TCGA: The Cancer Genome Atlas
GEO: Gene expression omnibus
RT-PCR: Reverse transcription-polymerase chain reaction
RT-qPCR: Real time-quantitative polymerase chain reaction
MVEs: multivesicular endosomes
SINEs: Short interspersed elements
