# Supplementary material for: Tumor-suppressive circRHOBTB3 is excreted out of cells via exosome to sustain colorectal cancer cell fitness
Source: Mol Cancer. 2022 Feb 11;21:46. doi: 10.1186/s12943-022-01511-1 (PMC8832727; doi:10.1186/s12943-022-01511-1)

A

| Series | total | type | sequencing platform |
|---|---|---|---|
| GSE100063 | 12 | CRC | Illumina HiSeq 2000 |
| GSE100207 | 21 | HCC | Illumina HiSeq 2000 |
| GSE100232 | 14 | PAAD | Illumina HiSeq 2000 |
| GSE100206 | 32 | Normal | Illumina HiSeq 2000 |

B

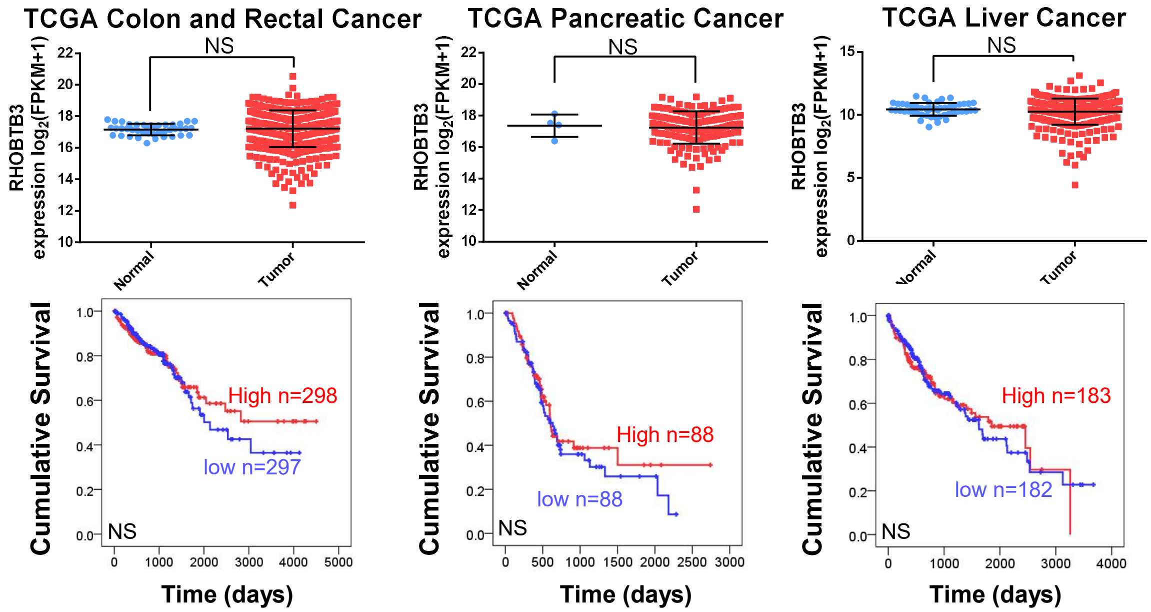

Supplement: Supplementary file 1 — Additional file 1: Fig. S1. Analysis of expression and survival from the TCGA and GEO databases. (A) The reanalyzed GEO datasets in this study. (B) Kaplan-Meier survival analysis of CRC, PAAD, and HCC patients according to the expression level of RHOBTB3 from the TCGA. All experiments were repeated for three times, data were shown as mean ± SD, NS P > 0.05, in Student’s test or log-rank test. [file 12943_2022_1511_MOESM1_ESM.pdf]
