# Supplementary figures and images for: Tumor-suppressive circRHOBTB3 is excreted out of cells via exosome to sustain colorectal cancer cell fitness
Source: Mol Cancer. 2022 Feb 11;21:46. doi: 10.1186/s12943-022-01511-1 (PMC8832727; doi:10.1186/s12943-022-01511-1)

A

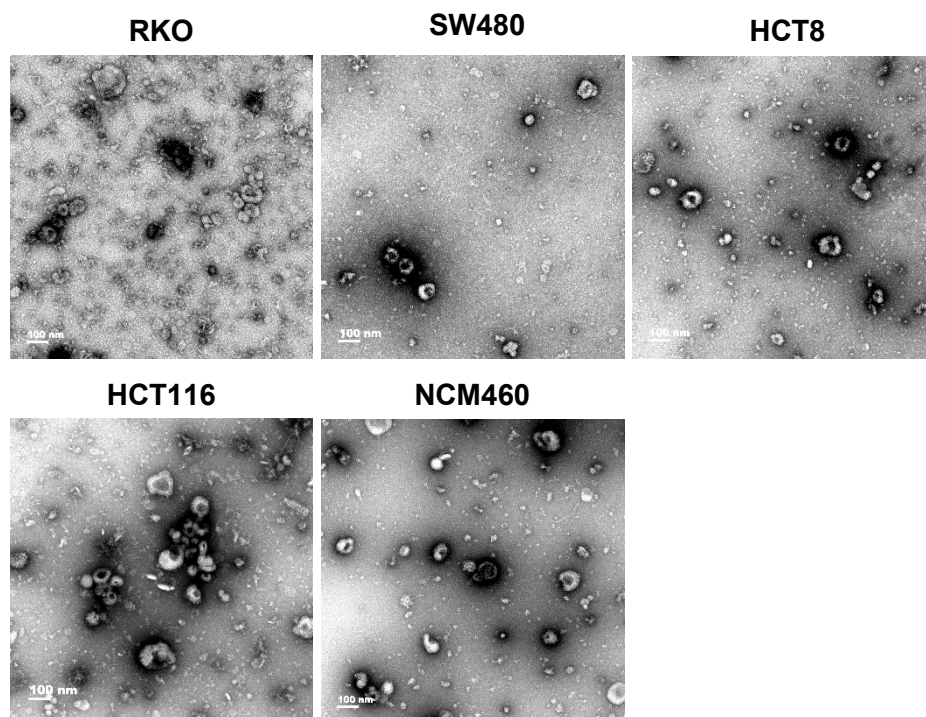

B

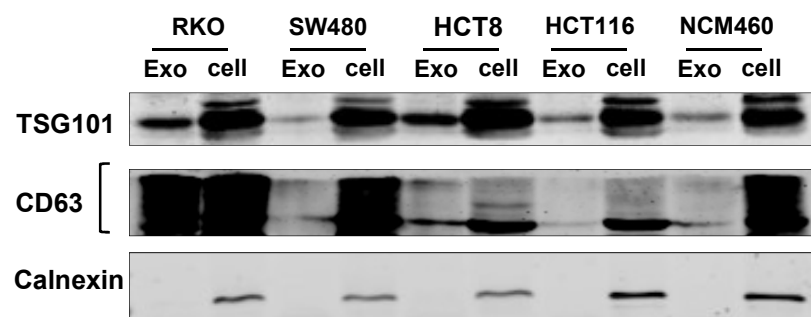

Supplement: Supplementary file 2 — Additional file 2: Fig. S2. Verification of exosomes. (A) Observation of exosomes by TEM. (B) Western blotting of CRC cell-derived exosomes using anti-CD63 and anti-TSG101 antibodies. Anti-calnexin was used as a negative control, and cellular lysates were used as positive loading controls. [file 12943_2022_1511_MOESM2_ESM.pdf]

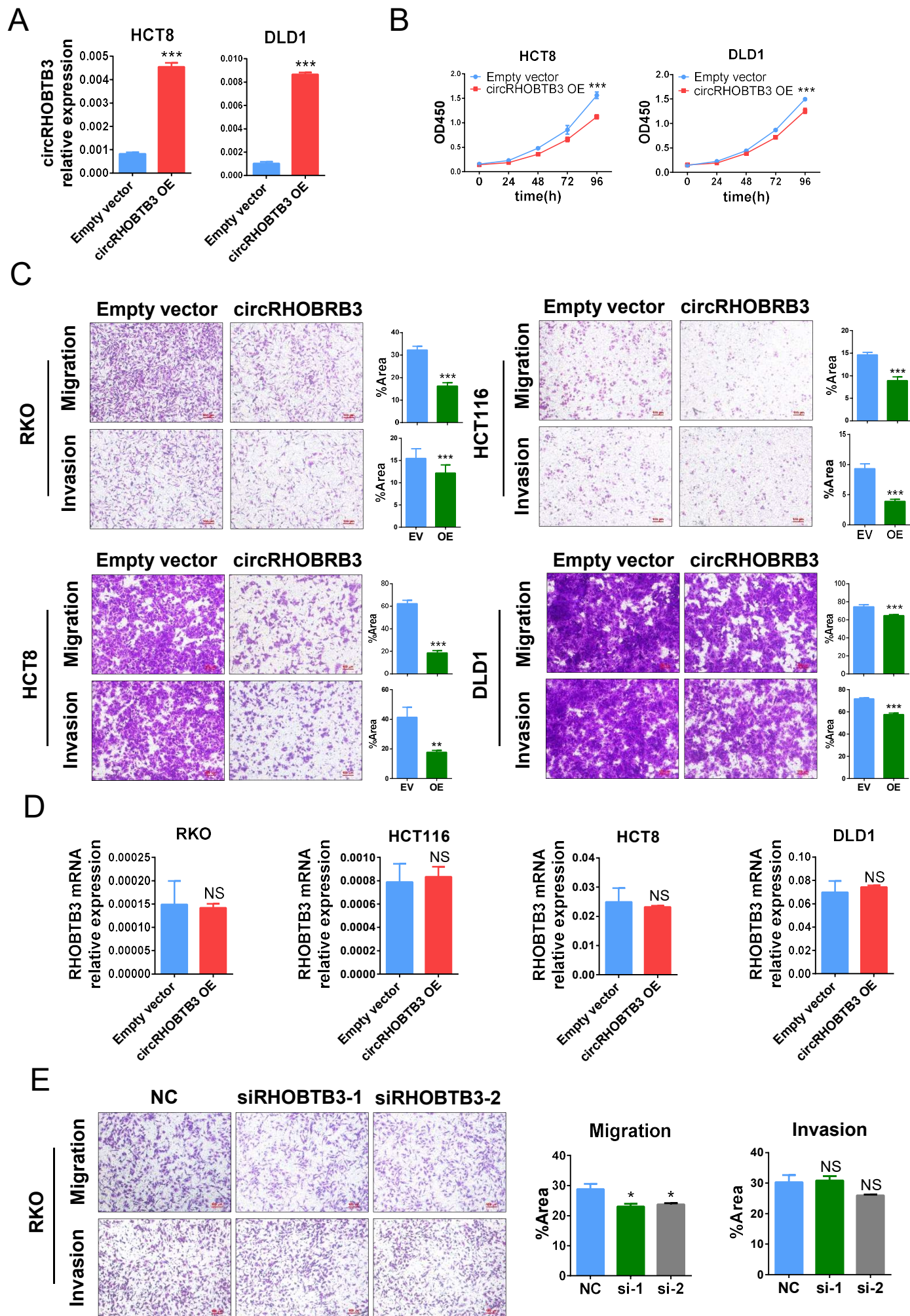

Supplement: Supplementary file 3 — Additional file 3: Fig. S3. circRHOBTB3 inhibits CRC cell proliferation, migration and invasion in vitro. (A) Overexpression of circRHOBTB3 via lentivirus vector transfection in HCT8 and DLD1 cells. (B) Cell proliferation of circRHOBTB3-OE HCT8 and DLD1 cells. (C) migration and invasion assays of circRHOBTB3-OE RKO, HCT116, HCT8 and DLD1 cells. The right column diagram shows the quantification analysis results. (D) Expression of RHOBTB3 in circRHOBTB3-OE RKO, HCT116, HCT8 and DLD1 cells. (E) Cell migration and invasion assay of circRHOBTB3-si RKO cells. The right column diagram shows the quantification analysis results. All experiments were repeated for three times, data were shown as mean ± SD (A, B, D) or mean ± SEM (C, E), * P < 0.05, ** P < 0.01, *** P < 0.001, NS P > 0.05, in Student’s test (A, B, D) or paired Student’s test (C, E). [file 12943_2022_1511_MOESM3_ESM.pdf]

A

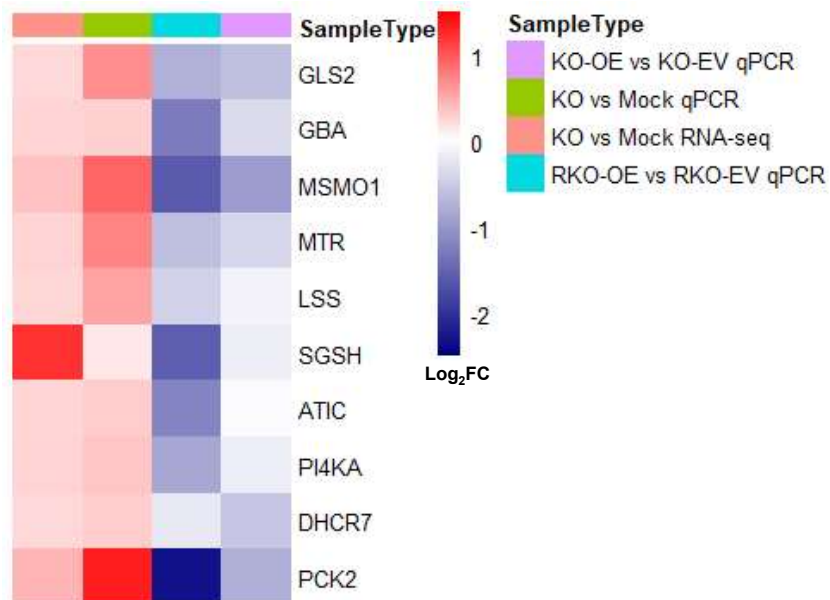

B

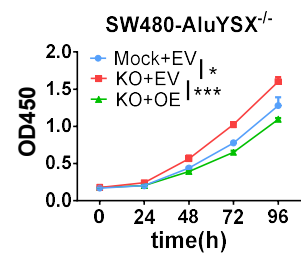

D

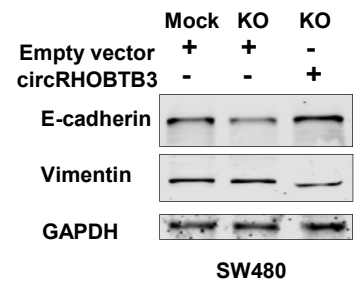

C

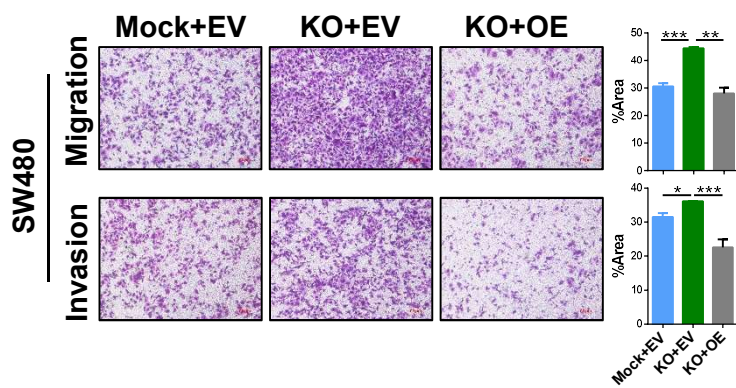

E

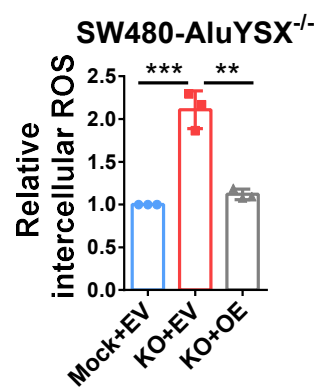

F

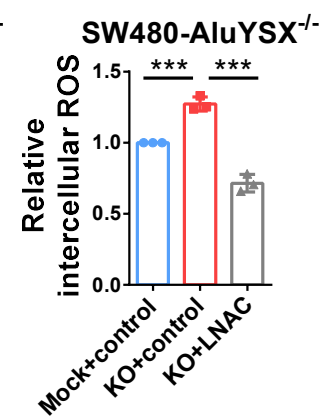

Supplement: Supplementary file 6 — Additional file 6: Fig. S6. circRHOBTB3 inhibits EMT through regulating ROS. (A) Heatmap of genes related to metabolism in circRHOBTB3-KO SW480 cells, circRHOBTB3-OE RKO cells, and circRHOBTB3-KO SW480 cells with circRHOBTB3 re-expression. (B) Cell proliferation, (C) migration and invasion assay and (D) Western blot of circRHOBTB3 overexpression in circRHOBTB3-KO SW480 cells. The right column diagram shows the quantification analysis results. (E) Relative intercellular ROS level of circRHOBTB3 re-expression and (F) LNAC-treated circRHOBTB3-KO SW480 cells. All experiments were repeated for three times, data were shown as mean ± SD (B) or mean ± SEM (C, E, F), * P < 0.05, ** P < 0.01, *** P < 0.001, NS P > 0.05, in Student’s test (B) or paired Student’s test (C, E, F). [file 12943_2022_1511_MOESM6_ESM.pdf]

A

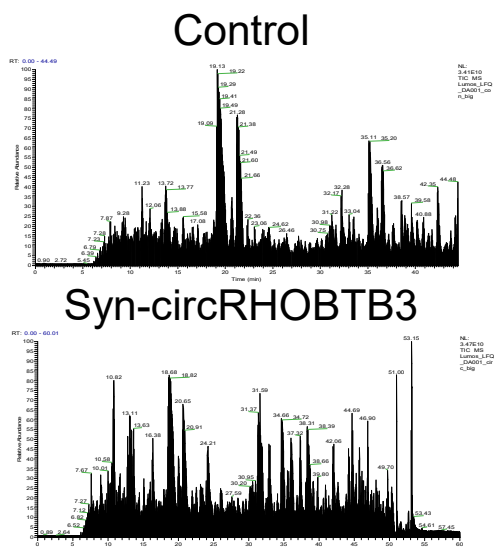

B

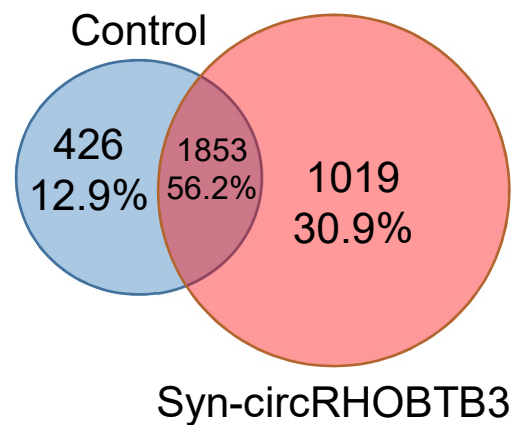

C

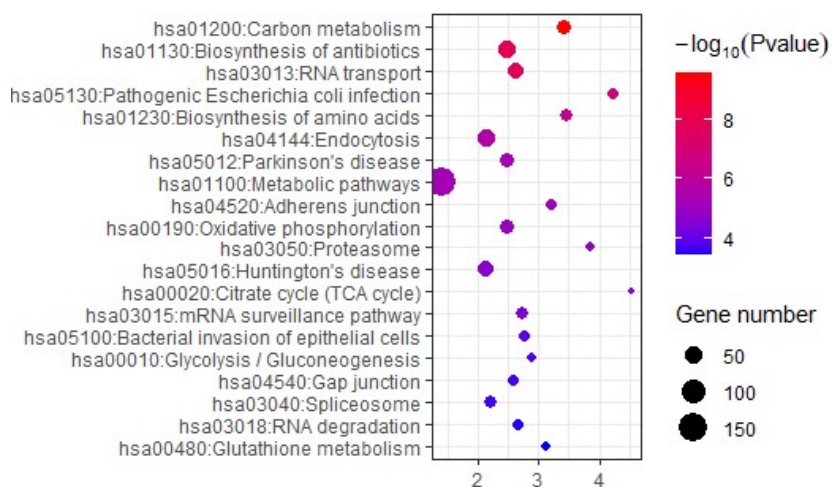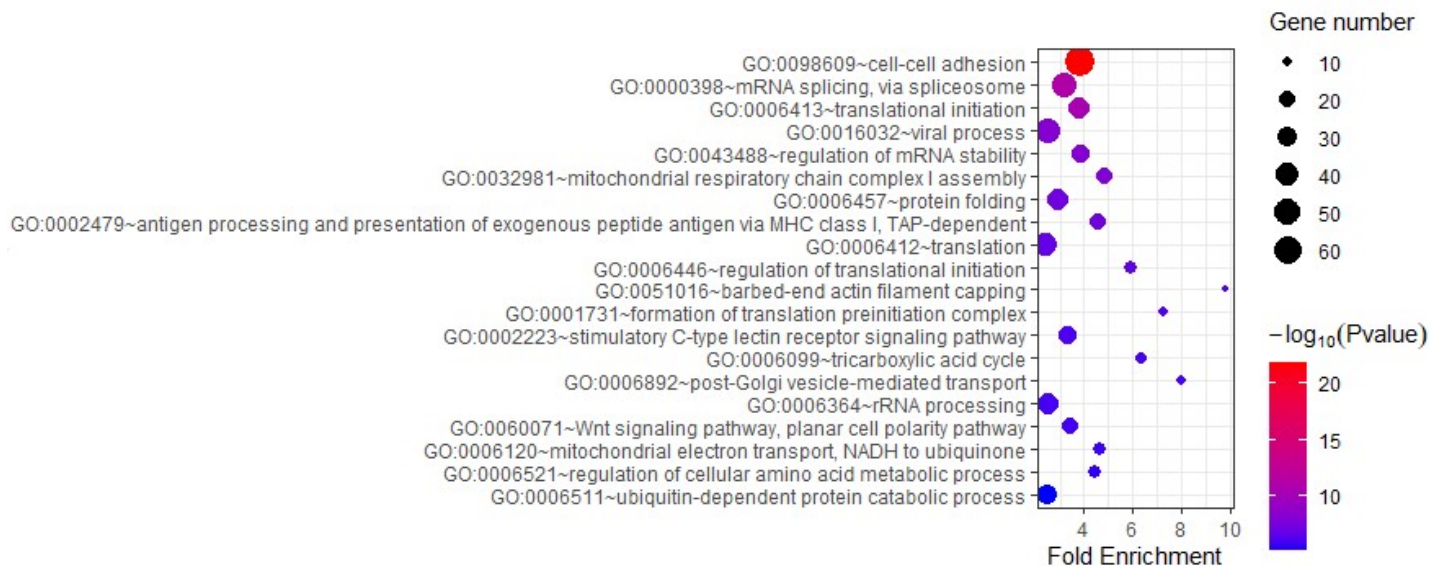

Supplement: Supplementary file 7 — Additional file 7: Fig. S7. Analysis of mass spectrometry results. (A) Total ion chromatogram (TIC) of control and syn-circRHOBTB3 pulldown products. (B) Venn diagram of proteins identified from control and syn-circRHOBTB3 pulldown products. (C) Enrichment analysis of potential proteins interacting with circRHOBTB3. [file 12943_2022_1511_MOESM7_ESM.pdf]

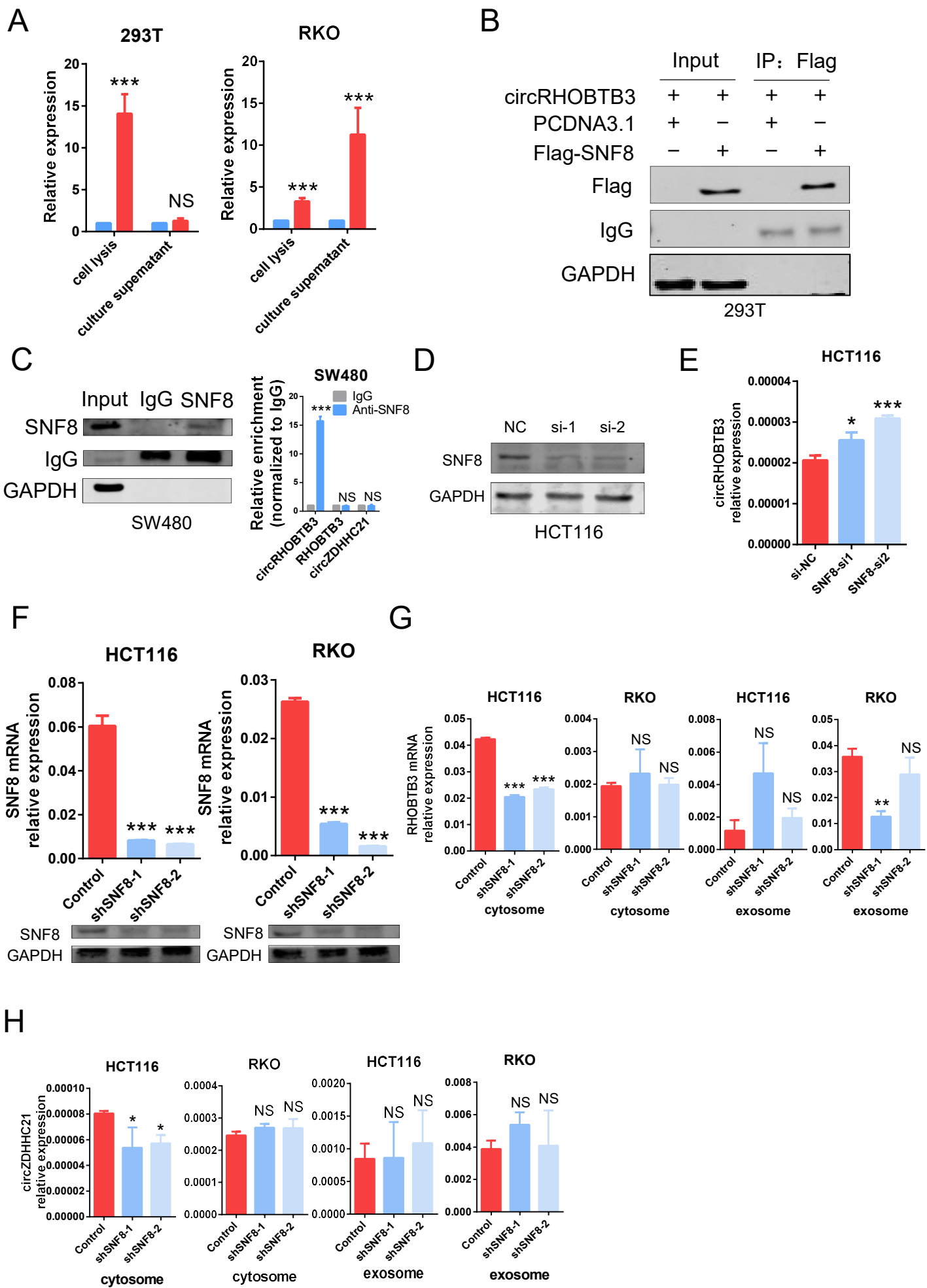

Supplement: Supplementary file 8 — Additional file 8: Fig. S8. SNF8 regulates the exosome sorting of circRHOBTB3. (A) Expression of circRHOBTB3 in cell lysis or culture supernatant of 293 T and RKO cells. (B) Western blot of Anti-FLAG RIP assay in 293 T cells. (C) Western blotting and RT-qPCR of the anti-SNF8 RIP assay in SW480 cells. (D) Western blotting of SNF8-KD HCT116 cells transfected with siRNA using anti-SNF8. (E) Expression of circRHOBTB3 in SNF8-KD HCT116 cells by siRNA. (F) RT-qPCR and Western blotting of SNF8-KD HCT116 cells transfected with shRNA. (G) Expression of circRHOBTB3 and (H) negative control circZDHHC21 in the cytosome and exosomes of SNF8-KD HCT116 and RKO cells. All experiments were repeated for three times, data were shown as mean ± SD (E, F, G, H) or mean ± SEM (A, C), * P < 0.05, ** P < 0.01, *** P < 0.001, NS P > 0.05, in Student’s test (E, F, G, H) or paired Student’s test (A, C). [file 12943_2022_1511_MOESM8_ESM.pdf]

A

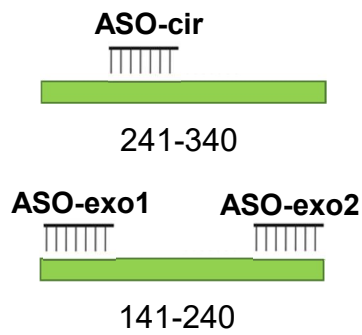

B

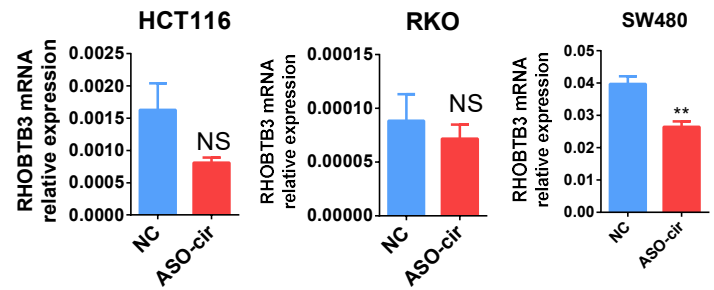

C

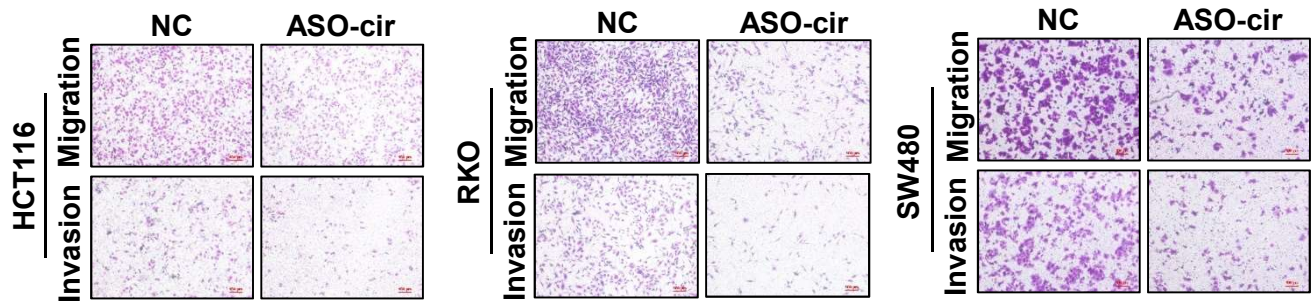

D

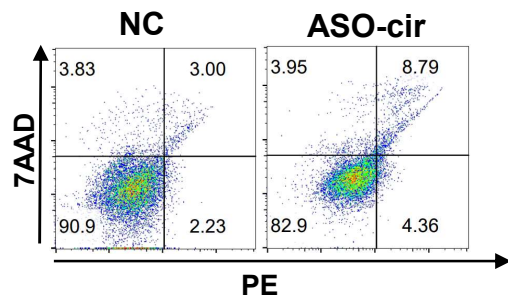

E

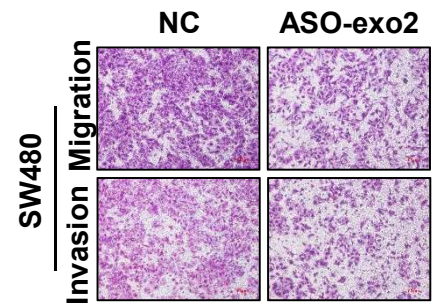

F

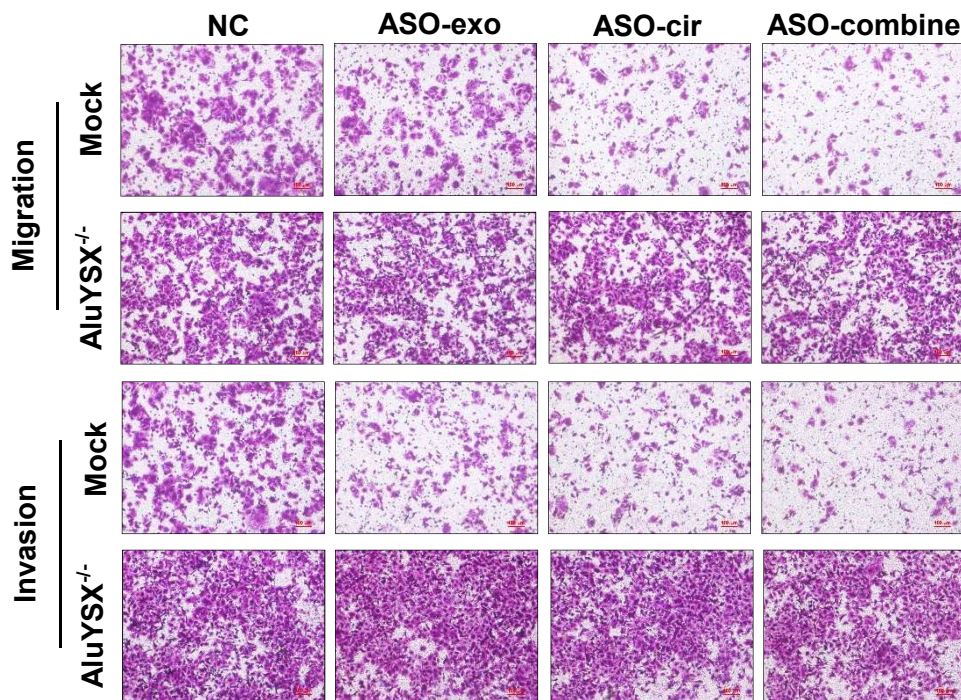

Supplement: Supplementary file 9 — Additional file 9: Fig. S9. ASO inhibited CRC cell proliferation, migration and invasion in vitro. (A) Schematic of ASO design. (B) Expression of RHOBTB3 in ASO-NC- and ASO-cir-treated HCT116, RKO and SW480 cells. (C) Cell migration and invasion assays of ASO-NC- and ASO-cir-treated HCT116, RKO and SW480 cells. (D) Apoptosis assay of ASO-NC- and ASO-cir-treated RKO cells. The right column diagram shows the quantification analysis results. (E) Cell migration and invasion assays of ASO-NC- and ASO-exo2-treated SW480 cells. (F) Cell migration and invasion assays of ASO-NC-, ASO-exo-, ASO-cir- and ASO-cir combined with ASO-exo-treated SW480 mock and KO cells. All experiments were repeated for three times, data were shown as mean ± SD (B) or mean ± SEM (D), * P < 0.05, ** P < 0.01, *** P < 0.001, NS P > 0.05, in Student’s test (B) or paired Student’s test (D). [file 12943_2022_1511_MOESM9_ESM.pdf]
