# Supplementary material for: Tumor-suppressive circRHOBTB3 is excreted out of cells via exosome to sustain colorectal cancer cell fitness
Source: Mol Cancer. 2022 Feb 11;21:46. doi: 10.1186/s12943-022-01511-1 (PMC8832727; doi:10.1186/s12943-022-01511-1)
Supplement: Supplementary file 4 — Additional file 4: Fig. S4. circRHOBTB3 is secreted outside of tumor cells and suppresses CRC cells in vitro and in vivo. (A) siRNA design for circRHOBTB3. (B) Expression of RHOBTB3 in circRHOBTB3-KD SW480 cells treated with RfxCas13d-BSJ-gRNA. (C) Migration and invasion assays of circRHOBTB3 KD SW480 cells. (D) Images of the subcutaneous xenograft tumors. (E) Images of the liver distant metastasis model established by splenic injection of control (empty vector) and circRHOBTB3-OE HCT116 cells in nude mice (the red arrows indicate metastatic foci). Data were shown as mean ± SD (B), NS P > 0.05, in Student’s test (B). [file 12943_2022_1511_MOESM4_ESM.pdf]

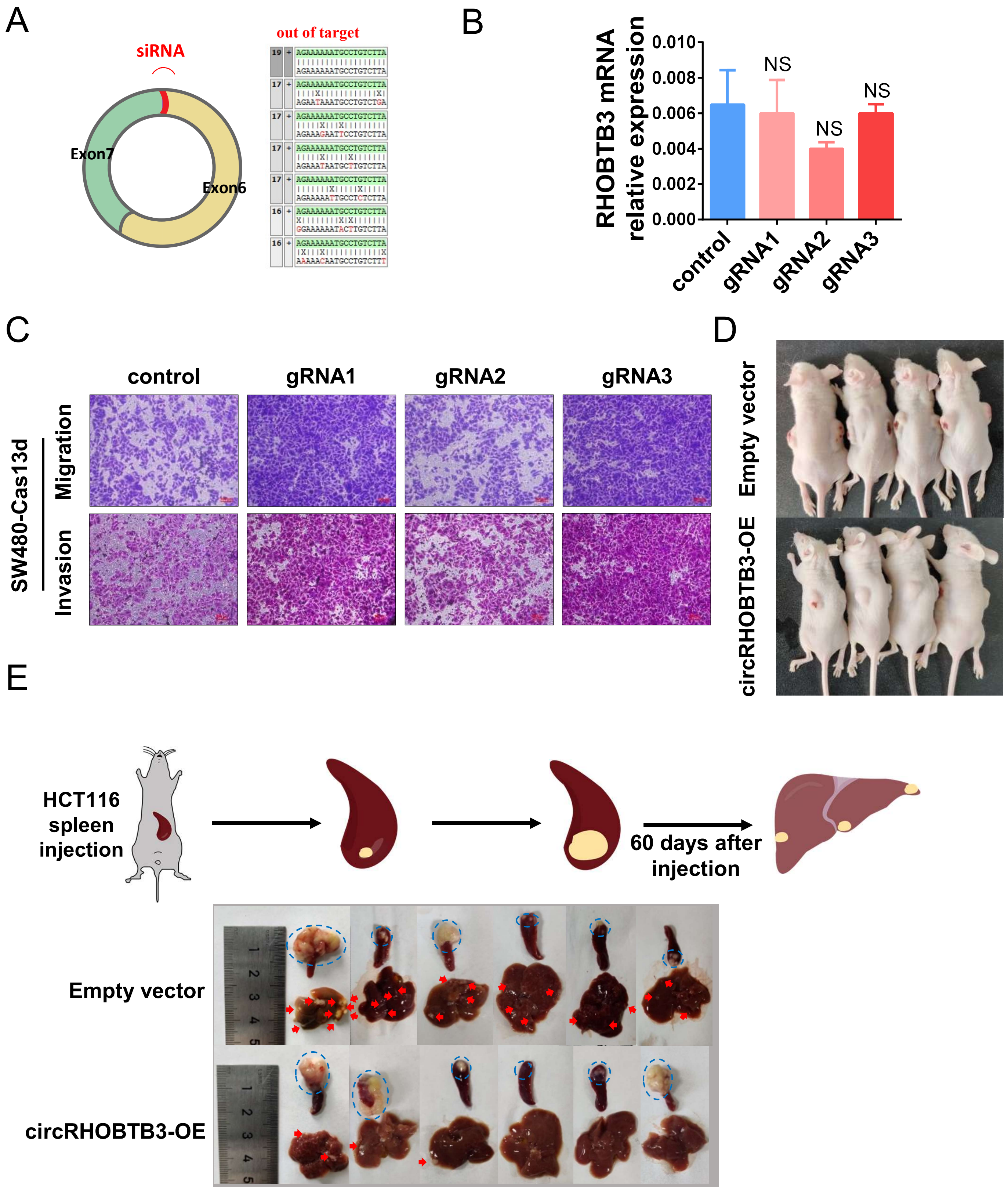

A
siRNA
Exon7
Exon6
out of target
B
RHOBTB3 mRNA
relative expression
0.010
0.008
0.006
0.004
0.002
0.000
NS
NS
NS
control
gRNA1
gRNA2
gRNA3
C
control
gRNA1
gRNA2
gRNA3
SW480-Cas13d
Migration
Invasion
D
Empty vector
circRHOBTB3-OE
E
HCT116
spleen
injection
60 days after
injection
Empty vector
circRHOBTB3-OE
