# Supplementary material for: Tumor-suppressive circRHOBTB3 is excreted out of cells via exosome to sustain colorectal cancer cell fitness
Source: Mol Cancer. 2022 Feb 11;21:46. doi: 10.1186/s12943-022-01511-1 (PMC8832727; doi:10.1186/s12943-022-01511-1)

A

IRES Elements		
Position	R Score	With Pseudoknot (Y/N)
266--412	1.611905	Y
271--405	1.582135	Y
Open Reading Frame (ORF)		
Start Position	End Position	Protein Length
455	2r+18	173 aa
MHFKNTRKEK MPVLKAEASH YNSDLNNLLF CCQCVDVVFY NPNLKKVVEA HKIVLCAVSH VFMLLFNVKS PTDIQDSSII RTTQDLFAIN RDTAFPGASH ESSGNPPLRV IVKDALFCSC LSDILRFIYS GAFQWEELEE DIRKKLKDSG DVSNVIEKVK CILKTPGKKK CLS*		
Note: (1). nr represents n rounds (n<3); (2). * represents a stop codon.		

B

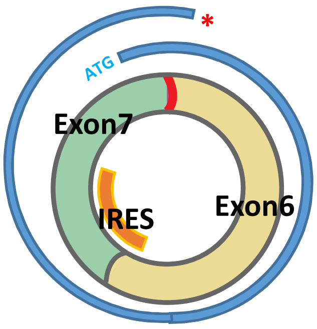

C

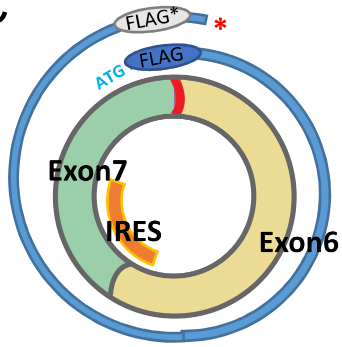

D

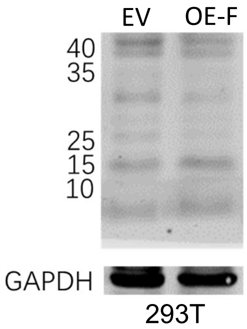

E

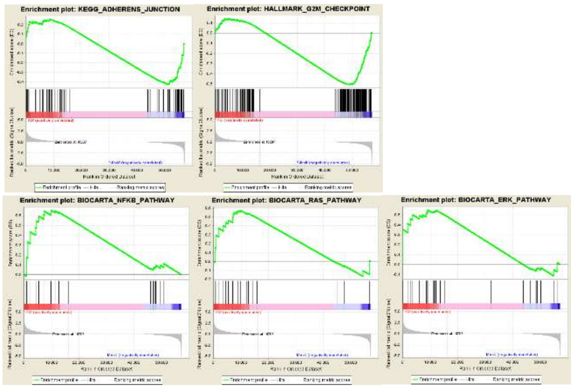

F

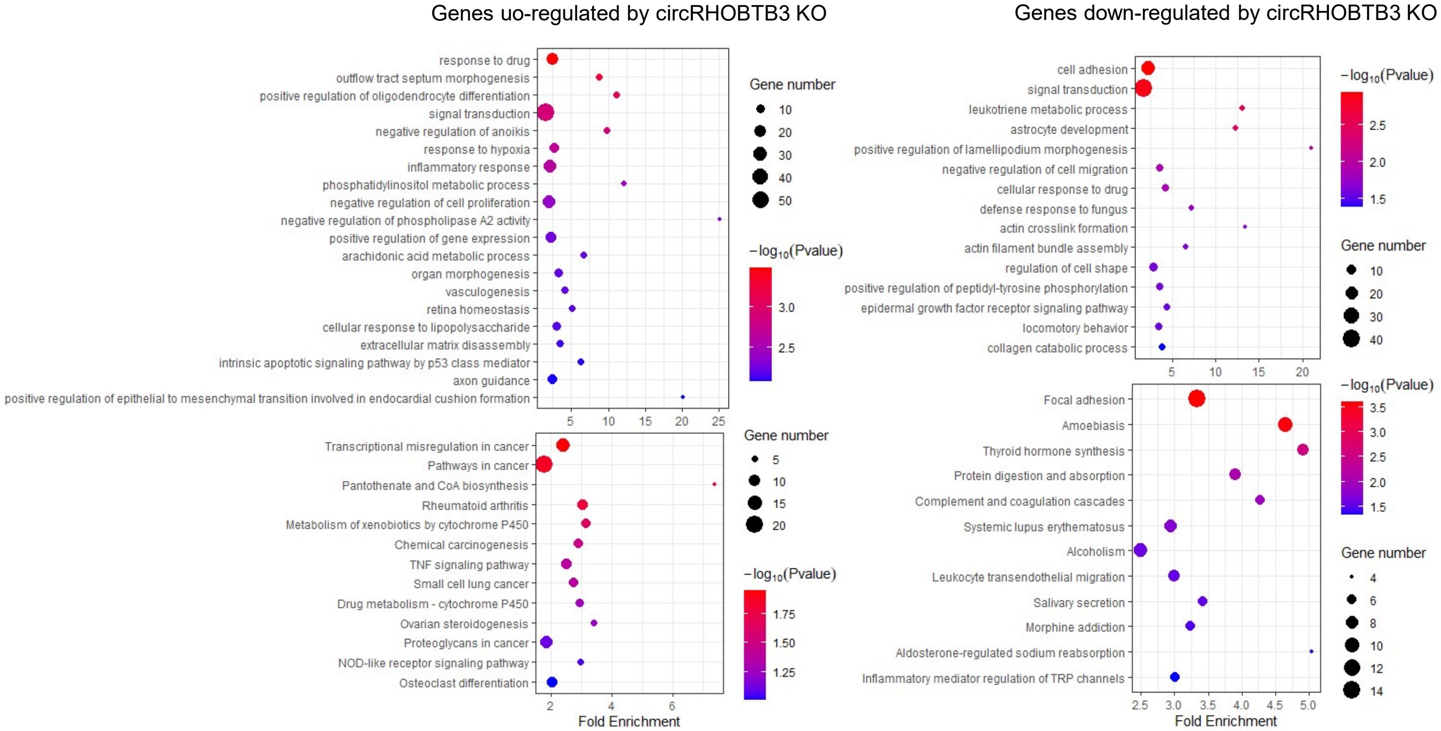

Supplement: Supplementary file 5 — Additional file 5: Fig. S5. The potential protein coding ability of circRHOBTB3. (A) IRES and ORF of circRHOBTB3. (B) Schematic of the potential protein encoded by circRHOBTB3. (C) Schematic of Flag-tagged circRHOBTB3 vector construction. (D) Western blotting of EV and Flag-tagged circRHOBTB3-OE 293 T cells using anti-Flag. (E) GSEA, (F) Kyoto Encyclopedia of Genes and Genomes (KEGG) and gene ontology (GO) enrichment analysis of circRHOBTB3-KO SW480 cells versus mock SW480 cells. [file 12943_2022_1511_MOESM5_ESM.pdf]
