# Supplementary material for: Tumor-suppressive circRHOBTB3 is excreted out of cells via exosome to sustain colorectal cancer cell fitness
Source: Mol Cancer. 2022 Feb 11;21:46. doi: 10.1186/s12943-022-01511-1 (PMC8832727; doi:10.1186/s12943-022-01511-1)
Supplement: Supplementary file 10 — Additional file 10: Table S1. Oligonucleotide sequences used in this study. Table S2. Vectors used in this study. Table S3. Antibodies used in this study. [file 12943_2022_1511_MOESM10_ESM.pdf]

Supplementary Materials and Methods

Public datasets analysis

The total-RNA expression profiling of serum exosomes from 12 CRC patients, 21 HCC patients, 32 PAAD patients, and 14 healthy donors of GSE100063, GSE100206, GSE100207, GSE100232 were obtained from GEO database. All these datasets were conducted on GPL11154 platform (Illumina HiSeq 2000). Processed data were downloaded from GEO and re-analyzed using MeV 4.9.0 software, $p<0.05$ and Foldchange>1.2 were considered statistically significant. The TCGA data were downloaded from The human protein atlas (https://www.proteinatlas.org/about/download).

Clinical materials

Colorectal carcinoma and paired normal tissues samples (n=35), another CRC cohort (n=69) were obtained from patients during operation at the Department of Medicine, Sir Run Run Shaw Hospital, Zhejiang University School of Medicine. 16 healthy donor serum samples and 18 CRC serum samples were obtained from CRC patients as well as medical examiners at the Department of Medicine (Ethics Committee number: 2021-021), Sir Run Run Shaw Hospital, Zhejiang University School of Medicine. All participating patients were informed. Patients or the public were not involved in the design, or conduct, or reporting, or dissemination plans of our research

Cell culture and cell lines

The human colorectal cancer RKO, HCT116, SW4810, HCT8, DLD1 cell line was purchased

from the American Type Culture Collection (ATCC, Manassas, VA, USA). 293T was purchased from the cell bank at the Chinese Academy of Sciences (Shanghai, China). The normal human colonic epithelial cell NCM460 was obtained from EK-Bioscience (shanghai, china). The normal human liver cell LO2 (HL-7702) was a gift from Q. Wang (The First Affiliated Hospital, Zhejiang University School of Medicine, China). RKO, 293T, LO2 was maintained in DMEM medium (Gibco). HCT116, SW4810, HCT8, DLD1, NCM460 was maintained in RPMI 1640 medium (Gibco). All media were supplemented with 10% (v/v) fetal bovine serum (Gibco) or 10% (v/v) exosome-free fetal bovine serum (for exosome isolation), glutamine and penicillin/streptomycin. All cell lines were maintained at 37°C, 5% CO2 in an incubator. Exosome-free FBS were prepared by ultracentrifugation according to Beckman official technical documentation (https://www.mybeckman.cn/support/technical-documents).

RNA isolation and RT-qPCR

Total RNA from cells or mice tissues was isolated using the Trizol reagent (Invitrogen, CAT#15596018). The exosomal total RNA and serum total RNA and mice plasma total RNA was isolated by using the Trizol LS (Invitrogen, CAT#10296010) and measured with Nanodrop 2000 (Thermo Scientific). For nuclear and cytoplasmic RNA fractionation, the PARIS Kit (Ambion CAT#AM1921) was used according to the manufacturer's instruction. The cDNA was synthesized by HiScript II Reverse Transcriptase (Vazyme, CAT#R223-01) and quantitative PCR analysis was performed by SYBR qPCR Master Mix (Vazyme, CAT#Q711-02).

RNase R digestion

Total RNA was incubated with or without 1U RNase R (Epicenter Biotechnologies) /1 μg RNA at 37℃ for 10 min. Finally, RNAs were analyzed by 2.0% TAE-agarose gel electrophoresis; Relative expression were determined using RT-qPCR, and relative ratios were calculated.

Transfection of ASOs and vectors

All ASOs, siRNA, shRNA, gRNA and primers are included in the Table S1, vectors are included in the Table S2. ASOs used *in vitro* and siRNAs were synthesized by GenePharma (Shanghai, china), ASOs used *in vivo* were synthesized by RiboBio (Guangzhou, China). Plasmids were transfected into cell lines by lipo2000 (Invitrogen, CAT#11668019), ASOs and siRNAs were transfected by GenMute siRNA transfection reagent (SignaGen, CAT#SL100568). All experiments were performed after 48h of transfection.

Cell proliferation, migration and invasion assay

2000 cells were seeded in 96-well plates and incubated with 100 μl culture medium, then were measured by CCK8 reagent (Boster, CAT#AR1160). Cell motility and invasiveness were measured by Transwell and matrigel chamber plates respectively (Corning Costar, CAT# 3422) as described before(Han et al., 2019). Image J 1.49v software was used to measure the cell area to quantify the cells migrated across the filter. Three fields were measured per Tanswell chamber and the experiments were repeated thrice.

Mouse models

Male nude mice with age 5 weeks were from SLAC LABORATORY ANIMAL (shanghai,

China) and used for all experiments, which were bred and maintained under SPF conditions at laboratory animal center, Zhejiang university. Experiments were performed in accordance with a protocol approved by the Institutional Animal Care and Use Committee at the Zhejiang University (Ethics Committee number: 19448).

For subcutaneously xenograft tumor models, 2×10^6 HCT116 EV or circRHOBTB3-OE cells were re-suspended in 100 µl PBS and injected into each flank of nude mice. Tumor growth was measured every day and tumor volume was calculated according to the following formula: length × width^2 × 0.5. All mice were euthanized at day 14 and tumors were separated and fixed in 10% buffered formalin, embedded in paraffin, sectioned and stained.

For liver metastasis models, 7×10^5 HCT116 EV or circRHOBTB3-OE cells were re-suspended in 100 µl PBS and inoculated into the spleen of nude mice. All mice were euthanized at day 60 and the spleens, livers, plasma, and urine were obtained. Spleens, livers were fixed in 10% buffered formalin, embedded in paraffin, sectioned and stained.

For ASO-injected models, 7×10^5 HCT116 cells were re-suspended in 100 µl PBS and inoculated into the spleen of nude mice. 20 nmol control or 10 nmol ASO-exo combined with 10 nmol ASO-cir were injected through the tail vein every three days. 30 days after inoculation, body weight was measured every 3 day after first injection. All mice were euthanized at day 60 and the spleens, livers, plasma, and urine were obtained. Spleens, livers were fixed in 10% buffered formalin, embedded in paraffin, sectioned and stained.

Histological analysis

Mice subcutaneous tumor, liver, and spleen samples were fixed with 10% formalin and paraffin

embedded. 4 μm thick sections were stained with hematoxylin and eosin (H&E) or processed for immunohistochemistry. Immunohistochemistry was performed as our previous described(Han et al., 2019). Primary antibodies are list in Table S3.

Isolation and identification of cellular exosomes

Cells cultured on 15 cm plates with medium containing 10% FBS were replaced with medium containing 10% exosome-free FBS when they reached 80% confluence and were maintained at 37°C, 5% CO_2 in an incubator until the cell medium was collected for exosome isolation after 48h. The cell medium was centrifuged at 200 g for 5 min to isolate live cells, followed by 1500 g for 10 min to remove cell debris and possible apoptotic bodies. Then, 14000 g for 70 min to extract microvesicies. After that, supernatant was filtrated with 0.22 um filter to remove any remaining MVs. The supernatant was ultracentrifuged at 170,000 g for 3 hours then resuspend pellet in PBS were ultracentrifuged at 170,000 g for 3 hours to isolate and wash exosomes. The exosomes were resuspended in 250 μl 1×phosphate-buffered saline (PBS) and verified with western blot and transmission electron microscope Tecnai G2 Spirit 120kV (FEI, Hillsboro, USA).

Immunoblotting

Whole cell lysates were extracted with RIPA and complete protease inhibitor mix (Fdbio science, CAT#FD009) followed by ultrasonication. Exosomes in PBS were lysed with 4×SDS buffer and complete protease inhibitor mix. Protein concentrations were measured by BCA assay (Thermo Scientific, CAT#23225). SDS-PAGE was performed to separated equal amounts

of protein lysates followed by transferring onto nitrocellulose membranes (Millipore), blocking with 5% skim milk and incubating with antibody overnight at 4 °C. Then membranes were incubated with fluorescence secondary antibodies for 1h in RT and detected by odyssey (LI-COR). All primary antibodies are list in Table S2.

Construction of knockout cell line

To construct circRHOBTB3-deficient SW480 cell, the pSpCas9-BB-2A vector containing sgRNA and pUC57 vectors containing homologous recombination donors was transfected to cells. 48h after transfection, 8 μg/ml puromycin were added into culture medium. After one week, puro-resistance sing cell was selected into a 96-well plate. These subclones were expanded and the knockout effect was examined by PCR and RT-qPCR in genomic and transcriptional level. The clones without genome editing were selected as mock cells. sgRNA sequences and PCR primers are list in Table S1.

Circularization efficiency assay

Full-length or truncated circRHOBTB3 expression vectors were transfected into 293T. 48h after transfected, total RNA were isolated and the cDNA was synthesized. The circularization efficiency of these truncated circRHOBTB3 was measured by RT-qPCR. To correct for differences in plasmid transfection, primers were designed in the sequence of CopGFP in pLCDH-ciR vector and used as reference to calculate the circularization efficiency.

RNA-seq, GSEA analysis and enrichment analysis

RNA of SW480-Mock and SW480-KO cells was extracted, sequenced and analyzed by Bioacme (Wuhan, China). Three biological replicates were used for condition. The individual RNA seq libraries were pooled based on their respective sample-specific-6 bp adaptors and sequenced at 150 bp/sequence pair-read using an Illumina NovaSeq system. Reads were mapping into the hg19 reference genome by STAR(Dobin et al., 2013) and quantified by RSEM(Li and Dewey, 2011). Gene differential expression analysis was accomplished by DESeq2(Love et al., 2014) package in R. Benjamini–Hochberg false discovery rate method was applied to correct for multiple hypothesis testing. The genes with adjust $P < 0.05$, fold change > 1.5 or < 0.67 was defined as different expression genes (DEGs) as candidates for further analysis. Gene expression heatmap was accomplished with R package pheatmap. Gene Ontology enrichment analysis was performed using online tools DAVID (http://david.abcc.ncifcrf.gov). The results were visualized by the R package ggplot2 in R software. Gene set enrichment analysis (GSEA) was performed with database MSigDBv7.2.

Cellular ROS assay and apoptosis assay using flow cytometer

For Cellular ROS assay, 0.3 million cells were seeded in 6-well plates. After 24h, cells were incubated with DCFH-DA (Beyotime, CAT#S0033S) at 37°C, 5% CO_2 in an incubator for 20 min. Cells were harvested in 1.5 ml tubes and washed three times with PBS followed by re-suspending in 300 µl of PBS. The cell suspension was filtered through cell strainer (40 µm nylon mesh) and examine the amount of ROS within cells by DxFLEX Flow Cytometer (Beckman, CA, USA). For apoptosis assay, 0.3 million cells were seeded in 6-well plates. After ASOs transfection for 48h, Annexin V-PE/7-AAD apoptosis kit (Multi sciences, Hangzhou,

China) were used to examine the amount of apoptosis ratio by DxFLEX Flow Cytometer.

Exosomal secretion assay

Full-length or truncated circRHOBTB3 expression vectors were transfected into cells. Total RNA from exosomes and cells were isolated 48h after transfected. The expression of circRHOBTB3 was measured by RT-qPCR and the exosomal secretion efficiency was calculated as

$$\textit{Exosomal secretion efficiency} = \frac{Exosome(Exp_{OE}/EXP_{EV})}{cytosome(Exp_{OE}/EXP_{EV})}$$

Biotin-labeled Circular RNA synthesis

The control was designed as reverse complementation of the circRHOBTB3 sequence. Linear transcription templates of control and circRHOBTB3 containing T7 promoter was constructed and amplified by PCR, The PCR product was purified by AxyPrep PCR cleanup kit (Axygen, CAT#AP-PCR-250). *In vitro* transcription was performed by RNAmax-T7 Biotin-labeled *in vitro* transcription Kit (Ribo, CAT#C11001-1) according to the manufacturer's instruction. To reduce the effect of biotin on the secondary structure of circRNA, we mixed unlabelled UTP (Ribo, CAT#C11002-1) with biotin-labelled UTP in a ratio of 1:5 (labelled:unlabelled). *In vitro* circularization of synthetic circRHOBTB3 was performed with reference to and improved upon previous reports(Liu et al., 2018). 20 μg linear RNA was treated with Quick CIP kit (NEB, CAT#M0525S) and then by T4 polynucleotide kinase (Thermo Scientific, CAT#EK0031) in the presence of ATP. Finally, the linear RNA was circularized using T4 RNA ligase 1 (NEB, CAT#M0204S). A control circRNA (described above) was also synthesized.

RNA pulldown, MS and ClueGO functional analysis

5 µg biotin-labeled synthetic circRHOBTB3 and antisense control was heated 90 °C and slow-cooled to 4 °C in annealing buffer for RNA Oligos (Beyotime, CAT#R0051), RNA was mixed with 1 mg SW480 cell lysis at 4 °C for 1h. Pre-washed Dynabeads M-280 streptavidin beads (Invitrogen, CAT#11206D) were added and further incubated at 4 °C overnight. Beads were collected by magnetic separator and washed briefly 6 times in DEPC-PBS and incubated at 100 °C in SDS buffer. SDS-PAGE was performed to separated pull-downed proteins followed silver-stained and MS to identify the proteins and then confirmed by western blot. The biological role of the circRHOBTB3-interacted 1019 genes were investigated in ClueGO. GO sets, MF, CC, BP, and KEGG pathways were used for the analysis.

RNA immunoprecipitation (RIP)

RIP was processed according to the protocol of the Magna RIP Kit (Millpore, CAT#17-700) except that the beads within the Kit was replaced by anti-M2 magnetic beads (Sigma-Aldrich, CAT#M8823) for exogenous RIP or SureBeads protein G magnetic beads (Bio-rad, CAT#1614023) and anti-SNF8 antibody (Table S2) for endogenous RIP to pull down the proteins with flag tag or SNF8 protein. Cell cultured in 15 cm plate was washed twice with 10 mL ice-cold DEPC-PBS and scraped off in 10 mL ice-cold DEPC-PBS, followed by centrifugation at 1500 rpm for 5 min at 4°C. The cell precipitation was re-suspended in 150 µl RIP Lysis Buffer. 50 µl M2 magnetic beads or 50 µl SureBeads protein G magnetic beads incubated with 10 µg anti-SNF8 antibody was washed twice with RIP Wash Buffer. 100 µl

Lysis and beads were mixed with 900 µl RIP Immunoprecipitation Buffer and incubated overnight at 4°C. Beads were collected by magnetic separator and washed by RIP Wash Buffer 6 times with briefly vortex. The immunoprecipitate was re-suspended in 150 µl proteinase K buffer and incubated at 55°C for 30 min in the hybrid furnace. Then, transfer the supernatant into 250 µl RIP Wash Buffer and 400 µl of Phenol:Chloroform:Isoamyl Alcohol (125:24:1) to each tubes. After centrifugation, the aqueous phase was transferred to a new tube and mixed with 400 µl of chloroform. Then, transfer the aqueous phase to another new tube with salt solution to enhance the precipitation of RNA overnight at -80°C. After centrifugation, the RNA is washed with 80% ethanol and resuspended in 15 µl of RNase-free water and measured with Nanodrop 2000.

Quantifications and statistical analysis

Data in this paper are presented as mean ± SD or mean ± SEM, and paired sample or two independent sample Student's t test was used to test for significant differences between two groups. Kaplan–Meier survival analysis was performed using the software IBM SPSS Statistics 20 with the Log-rank test. P-values <0.05 were considered statistically significant, *P < 0.05, **P < 0.01, ***P < 0.001, NS, not significant.

Reference

Dobin, A., Davis, C.A., Schlesinger, F., Drenkow, J., Zaleski, C., Jha, S., Batut, P., Chaisson, M., and Gingeras, T.R. (2013). STAR: ultrafast universal RNA-seq aligner. Bioinformatics *29*, 15–21.

Han, F., Zhang, L., Chen, C., Wang, Y., Zhang, Y., Qian, L., Sun, W., Zhou, D., Yang, B., Zhang, H., et al. (2019). GLTSCR1 Negatively Regulates BRD4-Dependent Transcription Elongation and Inhibits CRC Metastasis. Advanced Science *6*, 1901114.

Li, B., and Dewey, C.N. (2011). RSEM: accurate transcript quantification from RNA-Seq data with or without a reference genome. BMC Bioinformatics *12*, 323.

Liu, X., Abraham, J.M., Cheng, Y., Wang, Z., Wang, Z., Zhang, G., Ashktorab, H., Smoot, D.T., Cole, R.N., Boronina, T.N., et al. (2018). Synthetic Circular RNA Functions as a miR-21 Sponge to Suppress Gastric Carcinoma Cell Proliferation. Mol Ther Nucleic Acids *13*, 312–321.

Love, M.I., Huber, W., and Anders, S. (2014). Moderated estimation of fold change and dispersion for RNA-seq data with DESeq2. Genome Biol *15*, 550.

Supplementary Table S1. Oligonucleotide Sequences used in this study

**Crispr gRNA sequences**		
circRHOBTB3 KO gRNA1	TAATTGCAAAATCCACGTGAGGG	Cas9
circRHOBTB3 13d gRNA1	AAAACACCAGGAAAGAAAAAATGCCTGTCT	Cas13d
circRHOBTB3 13d gRNA2	ACCAGGAAAGAAAAAATGCCTGTCTTAAAG	Cas13d
circRHOBTB3 13d gRNA3	TTTTAAAAACACCAGGAAAGAAAAAATGCC	Cas13d
control	TCTGTCCGTAAAAAACTTTCCTGGTGTTTT	Cas13d
Direct repeat	gaaacaccgaacccctaccaactggtcggggtttgaaac	Cas13d-Direct repeat
**Genotyping Primer Sequences**		
Target	F	R
AluY	AGGAGAATGTTCCTGTTCCCAG	CAGGATCCTTTGGCTCTAAAAC
AluSx	ACCCTTGGGCCTTAAATCCT	CAGGATCCGTCCTTGGAACA
circRHOBTB3con	AAGATCGTTCTCTGCGCTGT	CTCGTAATGGTGGGTTGCCT
circRHOBTB3di	AGGCAACCCACCATTACGAG	ATGGCTTACAGCGCAGAGAA
**siRNA and shRNA**		
Target	Sense	Antisense
SNF8 si1	GCGGAAUACAGGAAGUAUATT	UAUACUUCCUGUAUUCCGCTT
SNF8 si2	GCUGAAGCGUCACAUUAUATT	UAUAAUGUGACGCUUCAGCTT
Negtive Control	UUCUCCGAACGUGUCACGUTT	ACGUGACACGUUCGGAGAATT
SNF8 sh1	CTGATAACTTTGGAGGAACTA	TAGTTCCTCCAAAGTTATCAG
SNF8 sh1	CGAACTAGGTGTCCAAATTAT	ATAATTTGGACACCTAGTTCG
**qPCR Primer Sequences**		
Target	F	R
circRHOBTB3con	AAGATCGTTCTCTGCGCTGT	CTCGTAATGGTGGGTTGCCT
circRHOBTB3di	AGGCAACCCACCATTACGAG	ATGGCTTACAGCGCAGAGAA
circRHOBTB3Δ	AAGTTAAATGCATTTTAAAAACACCAGG	ATAATGTGACGCTTCAGCCTT
lineRHOBTB3	TGCCTTTCAACCTGGCTACT	ACACCCACATTGGTTTCGGT
circZDHHC21	ACTCCCTGAGAACCCCAAGA	TGGGAACGCTTTGGTCTCAT
circCHD6	ATGGGATGGAGGACAGTGGA	TCTTTGGCTTGGGCTCTCTG
ACTB	GTCATTCCAAATATGAGATGCGT	GCTATCACCTCCCCTGTGTG
GAPDH	ACCACAGTCCATGCCATCAC	TCCACCACCCTGTTGCTGTA
MALAT1	TGAGGTGTTTGATGACCCGT	AACAACTCGCATCACCGGAA
SNF8	AGTATAAGGAGCGAGGGACGGT	ATCAGACCTCCATTCCGATGC
copGFP	GTGATCGGCGACTTCAAGGT	TGCTGCGGATGATCTTGTCG
GLS2	CCTGCGCTACAACAAGCTCT	GGAAAAATTACCTTCCGTGGC
GBA	TACTTGCAGGGCTAACCTAGT	GGGGGTCAAAGGAGTCACAG
MSMO1	TCGCGGCCGTTCAGGATAAG	CACAACCAAAGCATCTTGCCA
MTR	TCGCAACCCGAAGGTCTGAA	CCGGTAGGCCAAGTGTTCAA
LSS	CCTGTGCCATCTCCTACACG	TGAAGGCCATGGAACGCACA
SGSH	TGAGCCTCCTAGACCTCACG	TGGAAGGTGGGTGAGACGTA
ATIC	CGGCCAGCTCGCCTTATTTA	AGAGCTTTTGCAGTCCCTCC
PI4KA	GACGGTGGAGCAGAAATTTGG	CACATTGCGGATGGTTGCAT

DHCR7	ACTTTAGCCGGTTGAGAAGGA	CCCTTGAGATGCGGTTCTGT
PCK2	AGTGATGGTGGCGTGTACTG	CTGGGACTGGAAACTGCAAAC
**ASO**		
ASO-cir	GUUGCCTGAAGATTCATGGCUAGCA	
ASO-exo1	GCAUGAAAACATGGCTTACAGCGCA	
ASO-exo2	UAGCAAAAAGATCCTGGGTAGUUCG	

Supplementary Table S2. vector used in this study

Vectr name	Vector	Application
circRHOBTB3-OE	pLCDH-ciR	Overexpression
circRHOBTB3flag-OE	pLCDH-ciR	Overexpression
circRHOBTB3Δ41-140	pLCDH-ciR	Circulization efficiency and exosomal secretion assay
circRHOBTB3Δ141-240	pLCDH-ciR	Circulization efficiency and exosomal secretion assay
circRHOBTB3Δ241-340	pLCDH-ciR	Circulization efficiency and exosomal secretion assay
circRHOBTB3Δ341-440	pLCDH-ciR	Circulization efficiency and exosomal secretion assay
circRHOBTB3Δ241-265	pLCDH-ciR	Circulization efficiency assay
circRHOBTB3Δ266-290	pLCDH-ciR	Circulization efficiency assay
circRHOBTB3Δ291-315	pLCDH-ciR	Circulization efficiency assay
circRHOBTB3Δ316-340	pLCDH-ciR	Circulization efficiency assay
circRHOBTB3Δ141-165	pLCDH-ciR	exosomal secretion assay
circRHOBTB3Δ166-190	pLCDH-ciR	exosomal secretion assay
circRHOBTB3Δ191-215	pLCDH-ciR	exosomal secretion assay
circRHOBTB3Δ216-240	pLCDH-ciR	exosomal secretion assay
sh-sc	pLKO.1-TRC	control
shSNF8-1	pLKO.1-TRC	Knock down
shSNF8-2	pLKO.1-TRC	Knock down
BSJ-DR-control	pLKO.1-TRC	control
BSJ-DR-gRNA-1	pLKO.1-TRC	CRISPR/Cas13d
BSJ-DR-gRNA-2	pLKO.1-TRC	CRISPR/Cas13d
BSJ-DR-gRNA-3	pLKO.1-TRC	CRISPR/Cas13d
RfxCas13d	p23-NLS-RfxCas13d-mcherry-NLS-Flag	CRISPR/Cas13d
circRHOBTB3 KO gRNA1	pSpCas9-BB-2A	CRISPR/Cas9
ΔY-donor	pUC57	CRISPR/Cas9 donor
ΔSx-donor	pUC57	CRISPR/Cas9 donor
Flag-SNF8-OE	PCDNA3.1	RIP

Supplementary Table S3. Antibodies used in this study

Target	Clone	Company	Catologue Number	Application
TSG101	Polyclonal	Proteintech	28283-1-AP	WB
CD63	Polyclonal	Proteintech	25682-1-AP	WB
Calnexin	Monoclonal	Santa Cruz	sc-46669	WB
GAPDH	Monoclonal	Multi Science	Mab5456-040	WB
Vimentin	Monoclonal	CST	5741S	WB
E-cadherin	Monoclonal	CST	3195	WB
Eno1	Monoclonal	Santa Cruz	sc-100812	WB
Eno2	Monoclonal	Santa Cruz	sc-376375	WB
Flag	Monoclonal	Sigma-Aldrich	F1804	WB
SNF8	Monoclonal	Proteintech	67696-1-Ig	WB and RIP
Mouse IgG		Santa Cruz	sc-2025	RIP
Ki67	Polyclonal	Abcam	ab15580	Immunohistochemistry
